# Inequalities in cancer mortality trends in people with type 2 diabetes: 20 year population-based study in England

**DOI:** 10.1007/s00125-022-05854-8

**Published:** 2023-01-24

**Authors:** Suping Ling, Francesco Zaccardi, Eyad Issa, Melanie J. Davies, Kamlesh Khunti, Karen Brown

**Affiliations:** 1 Leicester Real World Evidence Unit, Leicester Diabetes Research Centre, Leicester General Hospital, University of Leicester, Leicester, UK; 2 Leicester Diabetes Research Centre, Leicester General Hospital, University of Leicester, Leicester, UK; 3 Present Address: Department of Non-communicable Disease Epidemiology, Faculty of Epidemiology and Population Health, London School of Hygiene & Tropical Medicine, London, UK; 4 National Institute for Health and Care Research (NIHR) Applied Research Collaboration East Midlands (ARC EM), University of Leicester, Leicester, UK; 5 Leicester HPB Unit, Leicester General Hospital, Leicester, UK; 6 Leicester Cancer Research Centre, Leicester Royal Infirmary, University of Leicester, Leicester, UK; 7 National Institute for Health and Care Research (NIHR) Leicester Biomedical Research Centre, University Hospitals of Leicester NHS Trust and the University of Leicester, Leicester, UK

**Keywords:** Cancer, Electronic health records, Inequalities, Mortality trends, Type 2 diabetes

## Abstract

**Aims/hypothesis:**

The aim of this study was to describe the long-term trends in cancer mortality rates in people with type 2 diabetes based on subgroups defined by sociodemographic characteristics and risk factors.

**Methods:**

We defined a cohort of individuals aged ≥35 years who had newly diagnosed type 2 diabetes in the Clinical Practice Research Datalink between 1 January 1998 and 30 November 2018. We assessed trends in all-cause, all-cancer and cancer-specific mortality rates by age, gender, ethnicity, socioeconomic status, obesity and smoking status. We used Poisson regression to calculate age- and calendar year-specific mortality rates and Joinpoint regression to assess trends for each outcome. We estimated standardised mortality ratios comparing mortality rates in people with type 2 diabetes with those in the general population.

**Results:**

Among 137,804 individuals, during a median follow-up of 8.4 years, all-cause mortality rates decreased at all ages between 1998 and 2018; cancer mortality rates also decreased for 55- and 65-year-olds but increased for 75- and 85-year-olds, with average annual percentage changes (AAPCs) of –1.4% (95% CI –1.5, –1.3), –0.2% (–0.3, –0.1), 1.2% (0.8, 1.6) and 1.6% (1.5, 1.7), respectively. Higher AAPCs were observed in women than men (1.5% vs 0.5%), in the least deprived than the most deprived (1.5% vs 1.0%) and in people with morbid obesity than those with normal body weight (5.8% vs 0.7%), although all these stratified subgroups showed upward trends in cancer mortality rates. Increasing cancer mortality rates were also observed in people of White ethnicity and former/current smokers, but downward trends were observed in other ethnic groups and non-smokers. These results have led to persistent inequalities by gender and deprivation but widening disparities by smoking status. Constant upward trends in mortality rates were also observed for pancreatic, liver and lung cancer at all ages, colorectal cancer at most ages, breast cancer at younger ages, and prostate and endometrial cancer at older ages. Compared with the general population, people with type 2 diabetes had a more than 1.5-fold increased risk of colorectal, pancreatic, liver and endometrial cancer mortality during the whole study period.

**Conclusions/interpretation:**

In contrast to the declines in all-cause mortality rates at all ages, the cancer burden has increased in older people with type 2 diabetes, especially for colorectal, pancreatic, liver and endometrial cancer. Tailored cancer prevention and early detection strategies are needed to address persistent inequalities in the older population, the most deprived and smokers.

**Graphical abstract:**

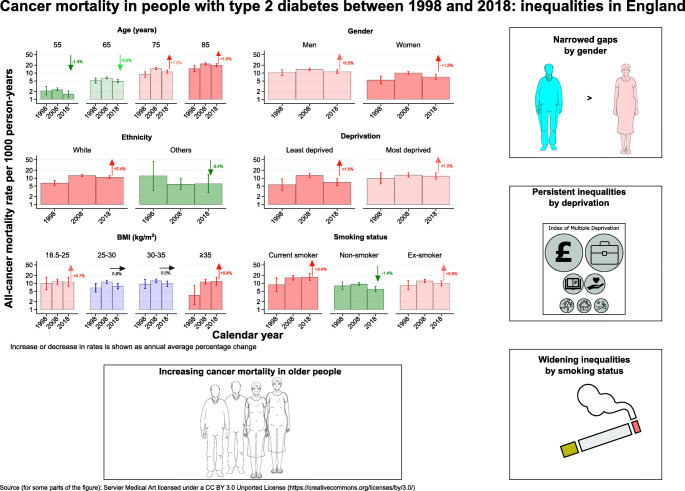

**Supplementary Information:**

The online version contains peer-reviewed but unedited supplementary material available at 10.1007/s00125-022-05854-8.

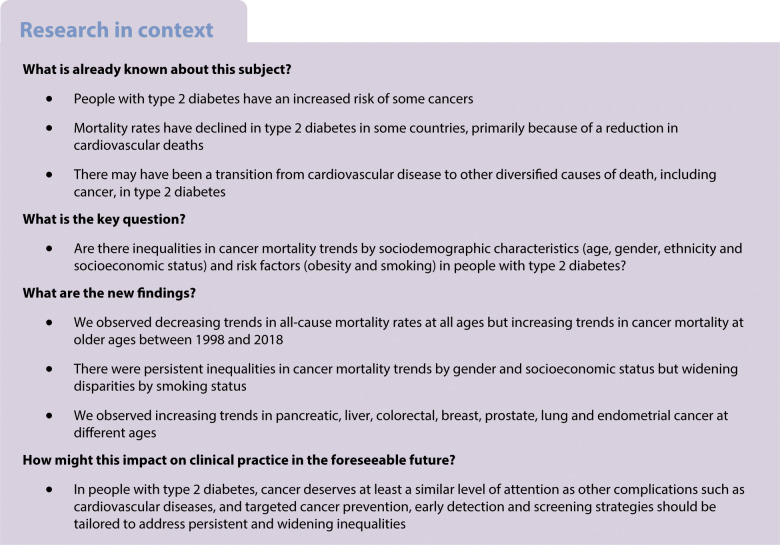



## Introduction

It was estimated that 537 million adults aged 20–79 years were living with diabetes worldwide in 2021, with more than 90% of them having type 2 diabetes [1]. Type 2 diabetes is associated with a higher risk of several vascular complications, including myocardial infarction, stroke, peripheral artery disease and kidney disease, leading to premature death [2]. Previous studies have reported declining rates in the last two decades of major cardiovascular complications and mortality in people with diabetes in some high-income countries [3, 4], with parallel greater contributions of other diseases, such as cancer, as the underlying causes of death [5]. Accumulating epidemiological evidence has indeed shown a higher risk of incidence and mortality for some types of cancer in individuals with type 2 diabetes [6, 7], with prolonged exposure to the effects of hyperglycaemia, hyperinsulinaemia, insulin resistance and chronic inflammation being the potential underlying biological mechanisms [6, 8]. Robust evidence indicates that there is a causal relationship between type 2 diabetes and pancreatic, liver and endometrial cancer [7]; both diabetes and cancer have also been linked to obesity and smoking [6, 8].

While previous studies have extensively investigated inequalities in vascular outcomes among people with type 2 diabetes by sociodemographic factors [9–12], less is known about whether such inequalities exist in cancer mortality rates. In this study we aimed therefore to describe long-term trends in cancer mortality rates in people with type 2 diabetes based on subgroups defined by sociodemographic characteristics and risk factors.

## Methods

### Data sources

We conducted this study following a prespecified research protocol, which was approved by the Clinical Practice Research Datalink (CPRD) Independent Scientific Advisory Committee (No. 19_120Mn), and the RECORD guidelines (see checklist in the electronic supplementary material [ESM]) [13].

We used the CPRD GOLD database to identify a cohort of individuals with type 2 diabetes in the UK. The CPRD routinely collects de-identified patient data, which are generally representative of the national population in terms of age, sex and ethnicity [14]. Data were linked to the Hospital Episode Statistics (HES), patient-level Index of Multiple Deprivation (IMD) 2010 and Office for National Statistics (ONS) death registrations (https://cprd.com/cprd-linked-data) to extract further information on ethnicity and hospitalisations, socioeconomic status and date and cause of death, respectively. Linkages were available only for patients in England.

### Study population

Individuals were included if they had a first-ever diagnosis code of type 2 diabetes in the CPRD between 1 January 1998 and 30 November 2018, were aged 35 years or over at the first diagnosis date of type 2 diabetes (i.e. the index date) and were registered with an up-to-standard practice for a minimum of 1 year at the index date. To rule out potential misclassification by clinical coding, individuals with a code of type 1 diabetes at any time in either the CPRD or HES were excluded. As the main outcome was cancer mortality, we only included individuals with linkage to ONS death registrations.

### Procedures

Individuals were categorised into subgroups defined by gender, ethnicity, socioeconomic status, BMI and smoking status. Ethnicity was grouped as White and other than White; data were predominantly extracted from HES and supplemented with CPRD records when data were missing in HES. We used the patient-level IMD 2010 quintiles (the mid-point year of the study period) to define socioeconomic status (1st quintile: least deprived; 5th quintile: most deprived). The IMD measures the relative deprivation for small areas in England and includes seven domains: income, employment, health and disability, education, skills and training, barriers to housing and other services, crime and living environment [15]. Data on BMI (underweight: <18.5; normal weight: 18.5–24.9; overweight: 25.0–29.9; obese: 30.0–34.9; and severely obese: ≥35.0 kg/m^2^) and smoking status (non-smoker, current smoker and ex-smoker) were extracted from the CPRD using the values for the closest date before the index date.

### Outcomes

The underlying cause of death was used to ascertain cancer deaths. To estimate the proportion of cancer deaths out of all-cause deaths, we also collected information on all-cause mortality. We further investigated deaths due to some specific cancers, including the four most common cancers in the UK (lung, colorectal, breast and prostate) and the four cancers causally linked to diabetes in a previous meta-analysis (i.e. pancreatic, liver, endometrial and gallbladder) [7]. All individuals were followed up from the index date until death or the end of study (linkage date for ONS data: 14 January 2019).

### Statistical analysis

We reported baseline characteristics (at the index date) as medians and IQRs for continuous variables and numbers and proportions for categorical variables; we also calculated person-years and numbers of events overall and in each subgroup. To estimate trends in mortality rates, we first split the risk time into 1 year intervals by attained age and attained calendar time and then modelled the outcomes with Poisson regressions including an interaction between a natural spline transformation of age and calendar year (five knots placed at the 10th, 30th, 50th, 70th and 90th percentile distribution in those with events) and adjusting for diabetes duration [16, 17]. Using log(person-time) as an offset, we predicted age-specific mortality rates at the mid-point of each calendar year and at the median diabetes duration on exiting the cohort (8.4 years). To further assess the cancer burden over time, we calculated the proportion of cancer deaths out of all-cause deaths and used non-parametric bootstrap sampling (500 samples with replacement) to derive 95% CIs. We conducted stratified analyses by gender, ethnicity, deprivation, BMI and smoking status to investigate potential inequalities. Individuals with missing data on ethnicity, socioeconomic status, BMI or smoking status were not included in the corresponding stratified analyses (<10% missingness). For stratified models, we predicted the mortality rates at the median age on exiting the cohort (72 years) to make rates comparable across subgroups. We then used the predicted rates to explore mortality trends and estimated the annual percentage changes (APCs) for each calendar year segment and the average annual percentage changes (AAPCs) for the whole study period using the Joinpoint Regression Program 4.9.1.0 [18]. We also calculated the age- and sex-standardised mortality ratios (SMRs) (age standardised only in sex-stratified analyses) for all outcomes by the calendar periods identified in the Joinpoint regressions, with corresponding mortality rates in the general population obtained from publicly available data in England and Wales [19]. Analyses were conducted in R 4.2.1 [20] (‘Epi’ package [16]), Joinpoint Regression Program 4.9.1.0 and Stata/BE 17.0 (StataCorp LLC, USA).

## Results

The study participant flow chart is provided in ESM Fig. 1. In total, 137,804 individuals were included in the analysis. Participant characteristics at type 2 diabetes diagnosis are shown in Table 1. The median age of participants was 63.8 years (IQR 54.2, 73.0); 61,444 (44.6%) were women; 114,394 (83.0%) were of White ethnicity; 64,652 (46.9%) were non-smokers; 16,126 (11.7%) had a normal body weight; and the median BMI was 30.6 kg/m^2^ (IQR 27.1, 34.9). During a median follow-up of 8.4 years (IQR 5.0, 12.2) and a total of 1,194,444 person-years, 39,212 (28.5%) deaths occurred. Table 2 reports the numbers of person-years and events stratified by each sociodemographic characteristic and risk factor.

**Table 1:** Baseline characteristics of individuals at type 2 diabetes diagnosis

Characteristic	Total (*N*=137,804)
Year of type 2 diabetes diagnosis	
1998	1804 (1.3)
1999	2277 (1.7)
2000	3592 (2.6)
2001	5278 (3.8)
2002	6710 (4.9)
2003	8225 (6.0)
2004	8705 (6.3)
2005	8822 (6.4)
2006	10,228 (7.4)
2007	8886 (6.4)
2008	9102 (6.6)
2009	9321 (6.8)
2010	9045 (6.6)
2011	8492 (6.2)
2012	8745 (6.3)
2013	8512 (6.2)
2014	6300 (4.6)
2015	5235 (3.8)
2016	3859 (2.8)
2017	2514 (1.8)
2018	2152 (1.6)
Age at diagnosis, years	
Median (IQR)	63.8 (54.2, 73.0)
<55	37,055 (26.9)
55–64.9	36,478 (26.5)
65–74.9	36,479 (26.5)
75–84.9	22,230 (16.1)
≥85	5562 (4.0)
Gender	
Men	76,360 (55.4)
Women	61,444 (44.6)
Ethnicity	
White	114,394 (83.0)
Other^a^	11,777 (8.5)
South Asian	4820 (3.5)
Black	2771 (2.0)
Other	4186 (3.0)
Missing	11,633 (8.4)
IMD 2010, quintiles	
1st (least deprived)	26,484 (19.2)
2nd	30,691 (22.3)
3rd	28,075 (20.4)
4th	28,198 (20.5)
5th (most deprived)	24,242 (17.6)
Missing	114 (0.1)
BMI, kg/m^2^	
Median (IQR)	30.6 (27.1, 34.9)
<18.5	579 (0.4)
18.5–24.9	16,126 (11.7)
25.0–29.9	43,026 (31.2)
30.0–34.9	37,203 (27.0)
≥35.0	31,262 (22.7)
Missing	9608 (7.0)
Smoking status	
Current smoker	23,044 (16.7)
Ex-smoker	45,616 (33.1)
Non-smoker	64,652 (46.9)
Missing	4492 (3.3)

Data are presented as *n* (%) unless indicated otherwise

^a^‘Other’ includes South Asian, Black and other ethnic groups

**Table 2:** Person-years and numbers of events at follow-up by gender, ethnicity, socioeconomic status, BMI group and smoking status

Characteristic	No. of individuals	Person-years	No. (%) of deaths^a^									
			All-cause	All-cancer	Type 2 diabetes-related cancer				Common cancer			
					Pancreatic	Liver	Gallbladder	Endometrial	Breast	Prostate	Colorectal	Lung
Total sample	137,804	1,194,444	39,212 (28.5)	11,309 (8.2)	1033 (0.7)	471 (0.3)	43 (0.0)	–	–	–	1093 (0.8)	2164 (1.6)
Gender	–											
Men	76,360	665,406	20,788 (27.2)	6613 (8.7)	536 (0.7)	328 (0.4)	11 (0.0)	–	–	848 (1.1)	647 (0.8)	1305 (1.7)
Women	61,444	529,038	18,424 (30.0)	4696 (7.6)	497 (0.8)	143 (0.2)	32 (0.1)	148 (0.2)	616 (1.0)	–	446 (0.7)	859 (1.4)
Ethnicity												
White	114,394	999,933	35,835 (31.3)	10,514 (9.2)	945 (0.8)	434 (0.4)	‡	‡	563 (0.5)	795 (0.7)	1015 (0.9)	2037 (1.8)
Other^b^	11,777	97,177	1128 (9.6)	338 (2.9)	30 (0.3)	26 (0.2)	<10	<10	29 (0.2)	31 (0.3)	30 (0.3)	43 (0.4)
South Asian	4820	41,827	392 (8.1)	107 (2.2)	<10	10 (0.2)	<10	<10	<10	<10	<10	<10
Black	2771	21,682	303 (10.9)	114 (4.1)	11 (0.4)	<10	<10	<10	‡	21 (0.8)	11 (0.4)	‡
Other	4186	33,668	433 (10.3)	117 (2.8)	‡	<10	<10	<10	‡	<10	‡	‡
IMD 2010, quintile												
1st (least deprived)	26,484	232,646	6833 (25.8)	2104 (7.9)	236 (0.9)	88 (0.3)	‡	33 (0.1)	118 (0.4)	184 (0.7)	198 (0.7)	321 (1.2)
2nd	30,691	269,942	8922 (29.1)	2691 (8.8)	261 (0.9)	115 (0.4)	<10	28 (0.1)	158 (0.5)	216 (0.7)	269 (0.9)	431 (1.4)
3rd	28,075	245,218	8169 (29.1)	2297 (8.2)	210 (0.7)	98 (0.3)	‡	28 (0.1)	124 (0.4)	183 (0.7)	227 (0.8)	430 (1.5)
4th	28,198	241,539	8064 (28.6)	2241 (7.9)	190 (0.7)	96 (0.3)	<10	36 (0.1)	126 (0.4)	156 (0.6)	213 (0.8)	471 (1.7)
5th (most deprived)	24,242	204,130	7172 (29.6)	1961 (8.1)	136 (0.6)	73 (0.3)	<10	22 (0.1)	96 (0.4)	108 (0.4)	185 (0.8)	509 (2.1)
BMI, kg/m^2^												
18.5–24.9	16,126	128,701	6821 (42.3)	1698 (10.5)	185 (1.1)	47 (0.3)	<10	11 (0.1)	89 (0.6)	130 (0.8)	137 (0.8)	360 (2.2)
25.0–29.9	43,026	376,630	12,982 (30.2)	3955 (9.2)	381 (0.9)	162 (0.4)	‡	31 (0.1)	185 (0.4)	334 (0.8)	381 (0.9)	759 (1.8)
30.0–34.9	37,203	327,139	8833 (23.7)	2846 (7.6)	239 (0.6)	135 (0.4)	‡	37 (0.1)	149 (0.4)	237 (0.6)	278 (0.7)	528 (1.4)
≥35.0	31,262	268,578	5674 (18.1)	1686 (5.4)	131 (0.4)	79 (0.3)	<10	53 (0.2)	126 (0.4)	77 (0.2)	156 (0.5)	301 (1.0)
Smoking status												
Current smoker	23,044	199,498	6789 (29.5)	2302 (10.0)	214 (0.9)	78 (0.3)	<10	<10	66 (0.3)	104 (0.5)	140 (0.6)	859 (3.7)
Ex-smoker	45,616	378,798	13,859 (30.4)	4262 (9.3)	358 (0.8)	170 (0.4)	‡	‡	175 (0.4)	374 (0.8)	414 (0.9)	955 (2.1)
Non-smoker	64,652	566,117	16,291 (25.2)	4261 (6.6)	427 (0.7)	204 (0.3)	‡	‡	352 (0.5)	339 (0.5)	480 (0.7)	280 (0.4)

Individuals with missing data on ethnicity, socioeconomic status, BMI or smoking status or individuals with underweight (BMI <18.5 kg/m^2^) were not included in the corresponding stratified analyses

^a^When the number of deaths was <10, full data are not shown for data protection reasons

^b^‘Other’ includes South Asian, Black and other ethnic groups

–, not applicable; ‡, the number of deaths was >10 but data are not shown to avoid disclosure of other cells <10

### All-cancer mortality rates

**Fig. 1:**
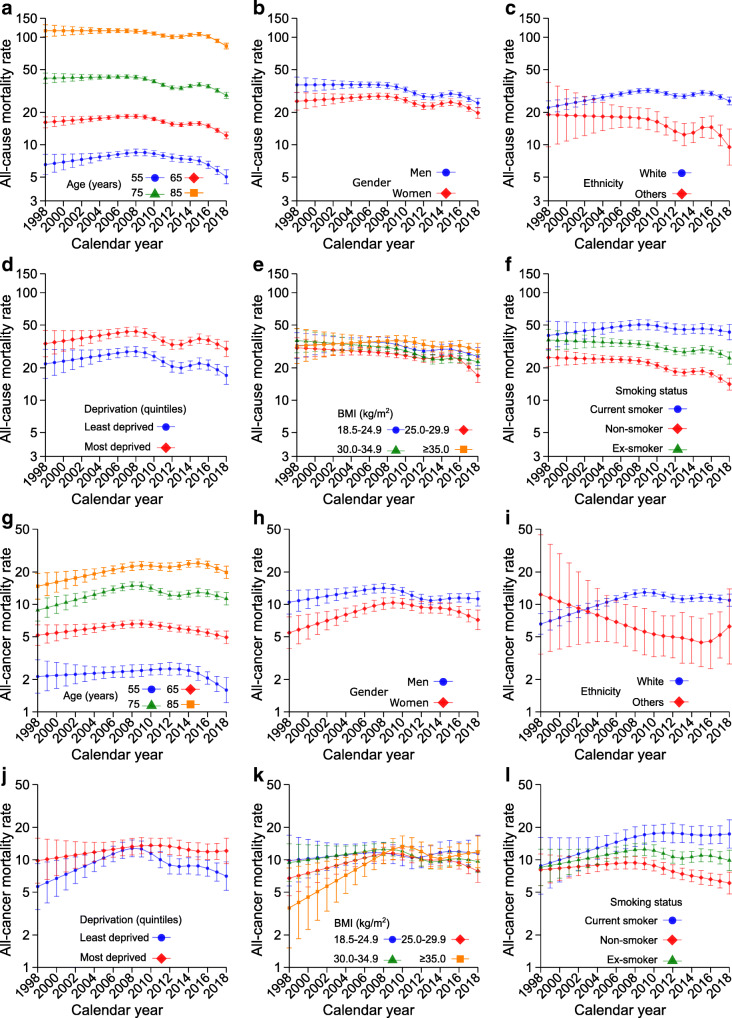
Trends in all-cause mortality rates (**a**–**f**) and all-cancer mortality rates (**g**–**l**) (per 1000 person-years). Age-specific mortality rates for all-cause mortality (**a**) and all-cancer mortality (**g**). All rates were estimated for the median diabetes duration at the end of follow-up (8.4 years). Rates stratified by gender (**b**, **h**), ethnicity (**c**, **i**), socioeconomic status (**d**, **j**), BMI (**e**, **k**) and smoking status (**f**, **l**) were also age-adjusted and are presented for the median age at the end of follow-up (72 years). Error bars indicate 95% CIs. The number of all-cancer deaths in people of ethnicities other than White was small in some years, leading to predicted rates with large uncertainties. All estimates are also reported in ESM Tables 1–6

**Fig. 2:**
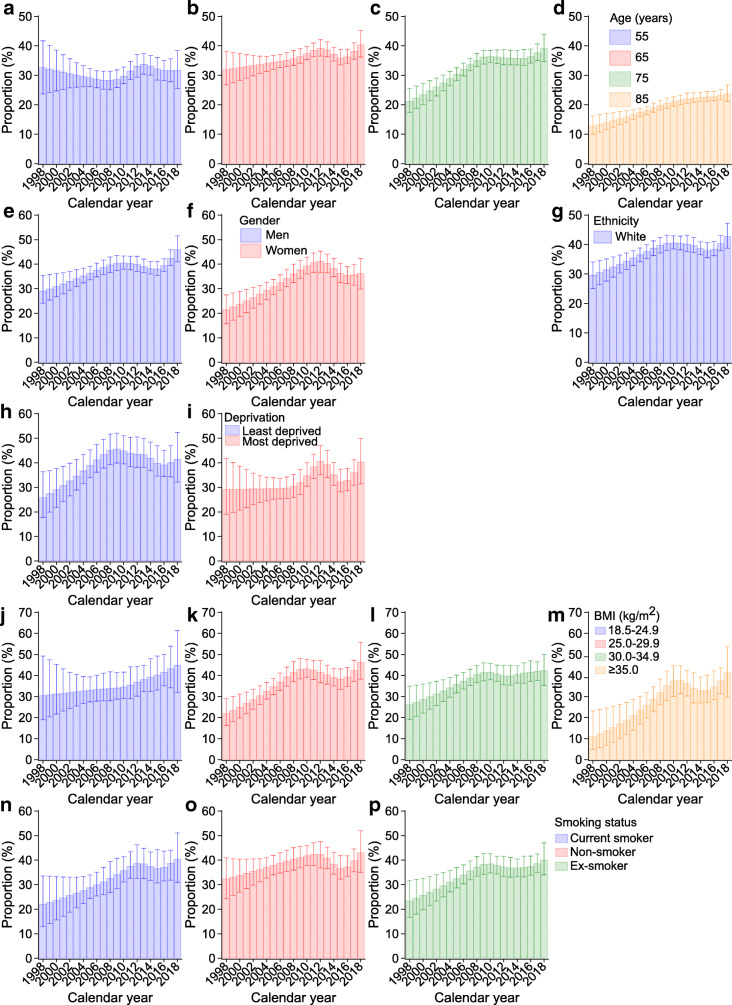
Proportions of cancer deaths out of all-cause deaths by age (**a**–**d**), gender (**e**, **f**), ethnicity (**g**), deprivation (**h**, **i**), BMI (**j**–**m**) and smoking status (**n**–**p**). Proportions were calculated as the all-cancer mortality rate divided by the all-cause mortality rate in each stratum and calendar year. Error bars indicate 95% CIs, which were estimated using the non-parametric bootstrap method with 500 replicates. The number of all-cancer deaths in people of ethnicities other than White was small in some years, leading to predicted rates with large uncertainties and unstable proportion estimates; only the proportions for White ethnicity are shown (**g**). All estimates are also reported in ESM Tables 1–6

Figure 1, ESM Tables 1–6 and Table 3 present trends in all-cause and all-cancer mortality rates in different subgroups. Trends in all-cause mortality rates are described in detail in the ESM (see Additional results: All-cause mortality rates). Figure 2 shows the proportions of cancer deaths out of all-cause deaths in different subgroups.

**Table 3:** APCs and AAPCs in all-cause and all-cancer mortality rates

Characteristic	Period 1		Period 2		Period 3		Period 4		AAPC for whole period (95% CI), %
	Years	APC (95% CI), %	Years	APC (95% CI), %	Years	APC (95% CI), %	Years	APC (95% CI), %	
All-cause deaths									
Age, years									
55	1998–2008	2.8 (2.5, 3.1)	2008–2015	–2.7 (–3.0, –2.4)	2015–2018	–9.9 (–11.6, –8.1)	–	–	–1.1 (–1.4, –0.8)
65	1998–2008	1.5 (1.4, 1.6)	2008–2012	–4.4 (–4.8, –3.9)	2012–2015	0.4 (–0.6, 1.4)	2015–2018	–7.3 (–8.0, –6.5)	–1.2 (–1.4, –1.0)
75	1998–2008	0.4 (0.2, 0.5)	2008–2012	–5.7 (–6.2, –5.3)	2012–2016	1.1 (0.5, 1.7)	2016–2018	–10.1 (–11.8, –8.3)	–1.8 (–2.1, –1.6)
85	1998–2008	0.0 (–0.1, 0.1)	2008–2012	–3.4 (–3.6, –3.1)	2012–2015	2.5 (1.9, 3.1)	2015–2018	–7.4 (–7.8, –7.0)	–1.5 (–1.6, –1.4)
Gender									
Men	1998–2008	0.0 (–0.1, 0.2)	2008–2012	–6.0 (–6.4, –5.5)	2012–2016	1.0 (0.4, 1.5)	2016–2018	–8.7 (–10.3, –7.0)	–1.9 (–2.1, –1.7)
Women	1998–2008	1.2 (1.1, 1.3)	2008–2012	–5.6 (–6.0, –5.1)	2012–2015	3.1 (2.1, 4.2)	2015–2018	–6.4 (–7.1, –5.7)	–1.1 (–1.3, –0.9)
Ethnicity									
White	1998–2009	3.4 (3.4, 3.5)	2009–2012	–4.5 (–5.1, –3.9)	2012–2016	1.5 (1.2, 1.9)	2016–2018	–8.1 (–9.1, –7.1)	0.6 (0.5, 0.8)
Other^a^	1998–2009	–0.8 (–0.8, –0.7)	2009–2013	–8.4 (–8.8, –8.0)	2013–2016	6.4 (5.3, 7.5)	2016–2018	–19.0 (–20.7, –17.3)	–3.3 (–3.5, –3.1)
Deprivation									
Least deprived	1998–2008	2.9 (2.6, 3.2)	2008–2013	–7.1 (–7.8, –6.5)	2013–2016	2.9 (0.3, 5.4)	2016–2018	–11.9 (–15.6, –8.0)	–1.3 (–1.8, –0.7)
Most deprived	1998–2008	2.9 (2.6, 3.1)	2008–2012	–7.2 (–7.8, –6.5)	2012–2016	2.7 (1.9, 3.6)	2016–2018	–9.9 (–12.3, –7.5)	–0.6 (–0.9, –0.3)
BMI group, kg/m^2^									
18.5–24.9	1998–2008	0.6 (0.5, 0.7)	2008–2012	–4.9 (–5.3, –4.6)	2012–2015	2.0 (1.2, 2.8)	2015–2018	–5.3 (–5.8, –4.7)	–1.2 (–1.4, –1.1)
25.0–29.9	1998–2008	–1.1 (–1.2, –1.0)	2008–2012	–3.1 (–3.4, –2.8)	2012–2015	2.7 (2.0, 3.3)	2015–2018	–11.8 (–12.3, –11.3)	–2.6 (–2.8, –2.5)
30.0–34.9	1998–2009	–1.5 (–1.5, –1.4)	2009–2013	–5.9 (–6.1, –5.7)	2013–2016	1.1 (0.6, 1.6)	2016–2018	–3.8 (–4.5, –3.0)	–2.2 (–2.3, –2.1)
≥35.0	1998–2010	0.9 (0.9, 1.0)	2010–2013	–4.5 (–4.9, –4.0)	2013–2016	0.8 (0.3, 1.4)	2016–2018	–5.3 (–6.0, –4.5)	–0.6 (–0.7, –0.4)
Smoking status									
Current smoker	1998–2008	2.4 (2.3, 2.5)	2008–2013	–2.3 (–2.5, –2.0)	2013–2016	0.4 (–0.4, 1.1)	2016–2018	–3.3 (–4.3, –2.3)	0.3 (0.2, 0.5)
Ex-smoker	1998–2009	–0.9 (–0.9, –0.8)	2009–2012	–4.8 (–5.1, –4.5)	2012–2016	0.7 (0.5, 0.9)	2016–2018	–8.1 (–8.7, –7.6)	–1.9 (–2.0, –1.8)
Non-smoker	1998–2008	–0.6 (–0.7, –0.5)	2008–2012	–6.0 (–6.4, –5.6)	2012–2015	0.5 (–0.4, 1.5)	2015–2018	–7.8 (–8.5, –7.1)	–2.6 (–2.8, –2.4)
All-cancer deaths									
Age, years									
55	1998–2012	1.3 (1.2, 1.3)	2012–2015	–2.6 (–3.1, –2.2)	2015–2018	–11.8 (–12.2, –11.3)	–	–	–1.4 (–1.5, –1.3)
65	1998–2006	2.6 (2.5, 2.7)	2006–2009	1.7 (1.0, 2.3)	2009–2015	–2.6 (–2.7, –2.5)	2015–2018	–4.4 (–4.8, –3.9)	–0.2 (–0.3, –0.1)
75	1998–2008	5.6 (5.3, 5.9)	2008–2013	–4.6 (–5.1, –4.1)	2013–2016	2.3 (0.6, 4.1)	2016–2018	–6.8 (–9.3, –4.2)	1.2 (0.8, 1.6)
85	1998–2008	4.5 (4.4, 4.6)	2008–2012	–0.7 (–1.0, –0.3)	2012–2015	3.3 (2.6, 4.0)	2015–2018	–6.1 (–6.6, –5.7)	1.6 (1.5, 1.7)
Gender									
Men	1998–2008	3.3 (3.0, 3.6)	2008–2013	–5.5 (–6.2, –4.8)	2013–2018	1.2 (0.5, 1.9)	–	–	0.5 (0.2, 0.8)
Women	1998–2008	6.7 (6.3, 7.2)	2008–2015	–2.0 (–2.3, –1.6)	2015–2018	–7.0 (–8.8, –5.1)	–	–	1.5 (1.1, 1.8)
Ethnicity									
White	1998–2009	6.3 (6.1, 6.4)	2009–2013	–4.3 (–4.8, –3.8)	2013–2016	1.4 (0.3, 2.5)	2016–2018	–3.1 (–4.6, –1.5)	2.4 (2.1, 2.6)
Other^a^	1998–2009	–7.1 (–7.2, –7.0)	2009–2016	–3.2 (–3.5, –2.9)	2016–2018	19.0 (16.4, 21.6)	–	–	–3.4 (–3.6, –3.2)
Deprivation									
Least deprived	1998–2008	8.9 (7.9, 9.9)	2008–2012	–8.7 (–10.9, –6.3)	2012–2018	–3.3 (–4.6, –1.9)	–	–	1.5 (0.7, 2.2)
Most deprived	1998–2009	3.0 (3.0, 3.0)	2009–2012	–0.5 (–0.8, –0.2)	2012–2015	–3.9 (–4.2, –3.6)	2015–2018	0.3 (0.0, 0.5)	1.0 (0.9, 1.1)
BMI group, kg/m^2^									
18.5–24.9	1998–2007	1.9 (1.8, 2.0)	2007–2012	–2.3 (–2.5, –2.1)	2012–2015	5.1 (4.5, 5.8)	2015–2018	–1.9 (–2.3, –1.5)	0.7 (0.6, 0.9)
25.0–29.9	1998–2008	5.6 (4.8, 6.3)	2008–2016	–2.6 (–3.2, –2.0)	2016–2018	–8.4 (–16.0, –0.1)	–	–	0.8 (–0.1, 1.7)
30.0–34.9	1998–2009	2.7 (2.6, 2.8)	2009–2013	–7.3 (–7.7, –6.9)	2013–2016	2.8 (1.8, 3.9)	2016–2018	–3.2 (–4.6, –1.8)	0.0 (–0.2, 0.3)
≥35.0	1998–2010	11.3 (10.9, 11.6)	2010–2014	–7.3 (–8.1, –6.6)	2014–2018	4.1 (3.4, 4.7)	–	–	5.8 (5.6, 6.1)
Smoking status									
Current smoker	1998–2008	6.5 (6.4, 6.6)	2008–2011	2.8 (2.6, 3.1)	2011–2015	–1.5 (–1.6, –1.4)	2015–2018	0.8 (0.6, 0.9)	3.4 (3.4, 3.5)
Ex-smoker	1998–2009	3.6 (3.5, 3.7)	2009–2013	–5.2 (–5.5, –4.9)	2013–2016	2.3 (1.5, 3.1)	2016–2018	–5.5 (–6.6, –4.5)	0.6 (0.5, 0.8)
Non-smoker	1998–2006	1.8 (1.8, 1.8)	2006–2009	0.1 (–0.1, 0.3)	2009–2013	–5.7 (–5.8, –5.6)	2013–2018	–3.8 (–3.8, –3.7)	–1.4 (–1.4, –1.3)

Segments (periods 1–4) were identified by Joinpoint regressions. Joinpoint regression was conducted for each stratum and therefore there could be different numbers of segments (from 1 to 4) for each stratum

^a^‘Other’ includes South Asian, Black and other ethnic groups

–, not applicable

Trends in and magnitudes of all-cancer mortality rates differed across age groups (Fig. 1g). During the whole study period, reductions were observed in younger age groups while increases were seen in older age groups: for 55-year-olds, the all-cancer mortality rate was 2.1 (95% CI 1.5, 3.1) per 1000 person-years in 1998, 2.4 (2.1, 2.7) in 2008 and 1.6 (1.2, 2.1) in 2018, with an AAPC of –1.4% (95% CI –1.5, –1.3) indicating an annual reduction of 1.4%. Corresponding rates and AAPCs were 5.2 (4.2, 6.4), 6.6 (6.0, 7.1), 4.9 (4.3, 5.7) and –0.2% (–0.3, –0.1), respectively, for 65-year-olds; 8.8 (7.0, 11.1), 14.9 (13.7, 16.2), 11.3 (9.9, 12.9) and 1.2% (0.8, 1.6), respectively, for 75-year-olds; and 14.7 (11.2, 19.4), 22.5 (20.6, 24.7), 19.8 (17.3, 22.6) and 1.6% (1.5, 1.7), respectively, for 85-year-olds (Fig. 1g; Table 3; ESM Table 1). Compared with other ages, the proportions of cancer deaths were noticeably lower but steadily increased in 85-year-olds during the whole study period (Fig. 2d; ESM Table 1). Proportions increased greatly in 75-year-olds but only slightly in 65-year-olds, followed by a flat trend after 2008 and 2012, respectively (Fig. 2b,c; ESM Table 1); proportions were constant for the youngest age group (Fig. 2a; ESM Table 1).

All-cancer mortality rates and proportions of cancer deaths were higher in men than women for most of the 1998–2018 period, with the gap in both rates and proportions being smaller around 2012–2014 and widening since 2014 (Figs 1h, 2e,f; ESM Table 2). AAPCs were 0.5% (95% CI 0.2, 0.8) in men and 1.5% (1.1, 1.8) in women (Table 3).

While all-cancer mortality rates increased before flattening and decreasing in people of White ethnicity, they decreased and then increased in people of other ethnicities, resulting in an overall increase in rate in people of White ethnicity (AAPC 2.4%; 95% CI 2.1, 2.6) and a reduction in people of other ethnicities (AAPC –3.4%; –3.6, –3.2) (Fig. 1i; Table 3; ESM Table 3). The proportion of cancer deaths increased slowly during the study period in people of White ethnicity (Fig. 2g) while it was not estimable in people with other ethnicities because of the small number of cancer deaths.

The deprivation gap in all-cancer mortality rates (most vs least deprived) was smallest around 2008 and widened thereafter (Fig. 1j; ESM Table 4). While the trends indicated increases in mortality rates in both the least and the most deprived groups from 1998 to 2018, the AAPC was slightly larger in the least deprived group (1.5%; 95% CI 0.7, 2.2) than in the most deprived group (1.0%; 0.9, 1.1) (Table 3). The proportions of cancer deaths increased from 1998 to 2008 in the least deprived group and from 2008 to 2013 in the most deprived group and then decreased and flattened in both groups; however, they were higher in the least deprived group than in the most deprived group for nearly all years during the study period (Fig. 2h,i).

All-cancer mortality rates at the beginning of the study were higher in people with normal weight than in people with overweight or morbid obesity. However, there were smaller increases in cancer mortality rates during the study period in individuals with normal weight (AAPC 0.7%; 95% CI 0.6, 0.9) than in those who were severely obese (AAPC 5.8%; 5.6, 6.1), resulting in no differences in cancer mortality rates between the two groups after 2012 (Fig. 1k; Table 3; ESM Table 5). During the entire study period we observed a sharply increasing trend in the proportions of cancer deaths in people who were overweight (Fig. 2k) or (severely) obese (Fig. 2l,m) but only a small increase in people with normal weight (Fig. 2j). Further, the proportions were similar in all BMI groups in 2018.

All-cancer mortality rates were appreciably higher in current than in former or non-smokers, particularly after 2008, with a progressively wider gap because of an increase in rates in current smokers (3.4%; 95% CI 3.4, 3.5) and former smokers (0.6%; 0.5, 0.8) but a reduction in non-smokers (–1.4%; –1.4, –1.3) (Fig. 1l; Table 3; ESM Table 6). The proportions of cancer deaths increased and then flattened in all three subgroups but such increases were sharper in current smokers (Fig. 2n) and former smokers (Fig. 2p) than in non-smokers (Fig. 2o); however, proportions across smoking groups were similar after 2010.

### Cancer-specific mortality rates

**Fig. 3:**
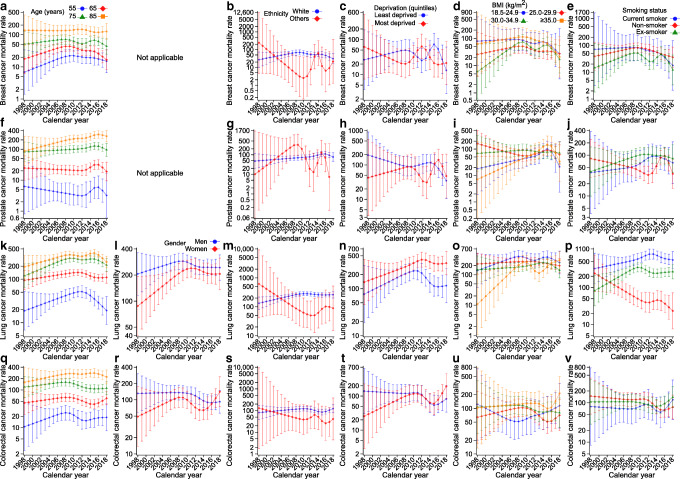
Trends in cancer-specific mortality rates (per 100,000 person-years) for four common cancers: (**a**–**e**) breast cancer, (**f**–**j**) prostate cancer, (**k**–**p**) lung cancer and (**q**–**v**) colorectal cancer. Age-specific mortality rates for breast (**a**), prostate (**f**), lung (**k**) and colorectal (**q**) cancer. All rates were estimated for the median diabetes duration at the end of follow-up (8.4 years). Rates stratified by gender (**l**, **r**), ethnicity (**b**, **g**, **m**, **s**), socioeconomic status (**c**, **h**, **n**, **t**), BMI (**d**, **i**, **o**, **u**) and smoking status (**e**, **j**, **p**, **v**) were also age-adjusted and are presented for the median age at the end of follow-up (72 years). Error bars indicate 95% CIs. Stratified analysis by gender is not applicable for breast and prostate cancer. The number of breast and prostate cancer deaths in people of ethnicities other than White was small in some years, leading to predicted rates with large uncertainties

**Fig. 4:**
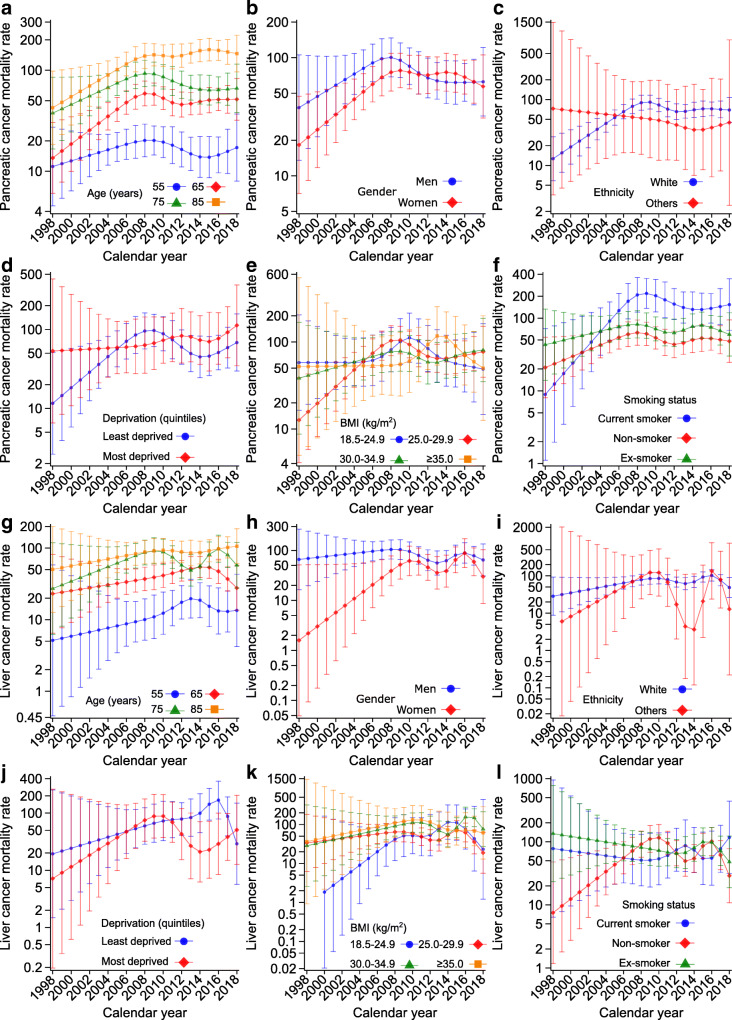
Trends in cancer-specific mortality rates for type 2 diabetes-related cancers (per 100,000 person-years). Age-specific mortality rates for pancreatic (**a**) and liver (**g**) cancer. All rates were estimated for the median diabetes duration at the end of follow-up (8.4 years). Rates stratified by gender (**b**, **h**), ethnicity (**c**, **i**), socioeconomic status (**d**, **j**), BMI (**e**, **k**) and smoking status (**f**, **l**) were also age-adjusted and are presented for the median age at the end of follow-up (72 years). Error bars indicate 95% CIs. The numbers of liver cancer deaths in people of ethnicities other than White and in the most deprived group were small in some years, leading to predicted rates with large uncertainties. Because of a small number of events, trends for gallbladder and endometrial cancer mortality rates are not shown but estimates are reported in ESM Table 8

Figure 3 shows the trends in mortality rates for breast, prostate, lung and colorectal cancer, the four most common cancers; ESM Table 7 reports the corresponding APCs and AAPCs by age, gender, ethnicity, socioeconomic status, BMI and smoking status. Of the four cancers causally linked to type 2 diabetes (pancreatic, liver, gallbladder and endometrial), Fig. 4 shows the trends in mortality rates only for pancreatic and liver cancer; because of the small number of events (Table 2), trends for gallbladder and endometrial cancer could not be estimated and stratified analyses were not possible. ESM Table 8 reports the corresponding APCs and AAPCs for all four cancers causally linked to type 2 diabetes by sociodemographic characteristics and risk factors.

Breast cancer mortality rates (Fig. 3a) increased slightly before decreasing in 55-, 65- and 75-year-olds and remained relatively stable in 85-year-olds, with AAPCs of 4.1% (95% CI 3.6, 4.7), –0.1% (–1.4, 1.2), –0.6% (–0.9, –0.3) and –0.5% (–0.5, –0.4), respectively (ESM Table 7). Prostate cancer mortality rates (Fig. 3f) increased in 75-year-olds (AAPC 0.8%; 0.6, 1.0) and 85-year-olds (5.6%; 5.5, 5.7) but decreased in 55-year-olds (–3.1%; –3.3, –2.8) and 65-year-olds (–1.2%; –1.4, –1.0) (ESM Table 7). Lung cancer mortality rates (Fig. 3k) increased slightly and then decreased for all age groups, with small increases from 1998 to 2018 in the two younger age groups (AAPCs 0.4% [0.2, 0.5] and 0.7% [0.6, 0.7] for 55- and 65-year-olds, respectively) and larger increases in the two older age groups (AAPCs 2.8% [2.5, 3.0] and 1.3% [1.1, 1.6] for 75- and 85-year-olds, respectively) (ESM Table 7). Except for those aged 75 years, colorectal cancer mortality rates (Fig. 3q) increased from 1998 to 2018 (AAPCs 3.1% [2.4, 3.9], 1.4% [1.2, 1.5], –0.5% [–0.7, –0.2] and 1.7% [1.6, 1.9] for 55-, 65-, 75- and 85-year-olds, respectively; ESM Table 7). Pancreatic cancer mortality rates (Fig. 4a) increased at all ages from 1998 to 2018 (AAPCs 2.0% [1.8, 2.2], 6.5% [6.0, 7.0], 2.6% [2.4, 2.9] and 6.1% [6.0, 6.3] for 55-, 65-, 75- and 85-year-olds, respectively; ESM Table 8). Steadily increasing trends were also observed for liver cancer mortality rates (Fig. 4g) at all ages (AAPC range 1.7–4.8%) and for endometrial cancer, except in the youngest age group (ESM Table 8).

Compared with women, men had higher lung, colorectal, pancreatic and liver cancer mortality rates but the gender gaps were smaller after 2010 and women had a higher colorectal cancer mortality rate than men after 2017 (Figs 3l,r, 4b,h; ESM Tables 7 and 8). We observed strong inequalities by socioeconomic status in lung cancer mortality rates, with markedly higher rates in the most deprived group (Fig. 3n). Lung cancer mortality rates were also higher in current smokers than in former or non-smokers (Fig. 3p), with increasing trends in current (AAPC 2.6%; 95% CI 1.9, 3.3) and former (5.7%; 5.5, 5.9) smokers and a decreasing trend in non-smokers (–11.0%; –11.1, –10.9) (ESM Table 7).

### Standardised mortality ratios

SMRs for all-cause, all-cancer and cancer-specific mortality comparing those with type 2 diabetes with the general population were estimated for the whole study period and three stratified periods: 1998–2007, 2008–2012 and 2013–2018 (Table 4). Individuals with type 2 diabetes had higher rates of all-cause, all-cancer and colorectal, pancreatic, liver, breast and endometrial cancer mortality, with SMRs for the whole period ranging from 1.08 to 2.40.

**Table 4:** SMRs comparing those with type 2 diabetes with the corresponding sex-specific general population

Cause of death	SMR (95% CI)^a^			
	1998–2007	2008–2012	2013–2018	Whole period
Overall				
All-cause	1.01 (0.99, 1.04)	1.19 (1.17, 1.21)	1.17 (1.15, 1.19)	1.08 (1.07, 1.09)
All-cancer	1.14 (1.09, 1.19)	1.26 (1.22, 1.30)	1.21 (1.18, 1.24)	1.18 (1.16, 1.20)
Common cancers				
Lung	0.87 (0.79, 0.97)	1.16 (1.08, 1.25)	1.08 (1.02, 1.15)	1.04 (1.00, 1.08)
Colorectal	2.10 (1.83, 2.39)	3.26 (2.93, 3.62)	2.81 (2.57, 3.06)	2.40 (2.26, 2.54)
Diabetes-related cancers				
Pancreatic	2.75 (2.41, 3.13)	2.36 (2.12, 2.62)	1.69 (1.54, 1.85)	2.12 (1.99, 2.25)
Liver	2.08 (1.60, 2.67)	1.85 (1.55, 2.20)	1.86 (1.65, 2.09)	2.13 (1.94, 2.33)
Gallbladder	1.26 (0.51, 2.60)	0.64 (0.24, 1.40)	1.77 (1.19, 2.52)	1.36 (0.99, 1.83)
Men				
All-cause	0.95 (0.92, 0.98)	1.14 (1.11, 1.17)	1.13 (1.11, 1.15)	1.02 (1.01, 1.04)
All-cancer	1.08 (1.02, 1.15)	1.15 (1.10, 1.21)	1.14 (1.10, 1.18)	1.09 (1.06, 1.12)
Common cancers				
Prostate	0.85 (0.71, 1.01)	0.98 (0.86, 1.11)	1.11 (1.02, 1.22)	0.99 (0.92, 1.05)
Lung	0.78 (0.68, 0.89)	1.03 (0.93, 1.13)	1.03 (0.95, 1.11)	0.91 (0.86, 0.96)
Colorectal	2.03 (1.71, 2.40)	2.73 (2.37, 3.13)	2.36 (2.11, 2.64)	2.10 (1.94, 2.27)
Diabetes-related cancers				
Pancreatic	2.77 (2.31, 3.28)	2.11 (1.82, 2.44)	1.47 (1.28, 1.67)	1.90 (1.75, 2.07)
Liver	2.35 (1.73, 3.12)	1.85 (1.49, 2.28)	1.83 (1.58, 2.11)	2.12 (1.90, 2.36)
Gallbladder	1.49 (0.31, 4.36)	0.65 (0.08, 2.34)	1.07 (0.39, 2.34)	1.00 (0.50, 1.79)
Women				
All-cause	1.03 (0.99, 1.06)	1.20 (1.17, 1.23)	1.16 (1.14, 1.19)	1.09 (1.08, 1.11)
All-cancer	1.14 (1.07, 1.22)	1.34 (1.27, 1.41)	1.22 (1.18, 1.28)	1.22 (1.19, 1.26)
Common cancers				
Breast	1.07 (0.89, 1.27)	1.11 (0.96, 1.29)	1.20 (1.07, 1.34)	1.09 (1.01, 1.18)
Lung	0.95 (0.79, 1.13)	1.29 (1.15, 1.44)	1.08 (0.98, 1.19)	1.15 (1.07, 1.23)
Colorectal	1.98 (1.57, 2.46)	3.86 (3.25, 4.54)	3.29 (2.87, 3.76)	2.61 (2.38, 2.87)
Diabetes-related cancers				
Pancreatic	2.62 (2.14, 3.19)	2.59 (2.23, 3.00)	1.92 (1.67, 2.19)	2.30 (2.11, 2.51)
Liver	1.29 (0.70, 2.16)	1.63 (1.18, 2.21)	1.65 (1.32, 2.04)	1.82 (1.54, 2.15)
Gallbladder	1.19 (0.32, 3.05)	0.68 (0.19, 1.74)	2.28 (1.46, 3.39)	1.68 (1.15, 2.36)
Endometrial	1.97 (1.24, 2.99)	2.18 (1.62, 2.88)	1.75 (1.38, 2.19)	2.08 (1.76, 2.44)

SMRs were estimated for all-cause mortality, all-cancer mortality and cancer-specific mortality for the four most common cancers and the four type 2 diabetes-related cancers

^a^Age- and sex-standardised mortality ratios for the overall population (men and women); age-standardised mortality ratios for sex-specific estimates

SMRs for all-cause and all-cancer mortality increased and then levelled off after the 2008–2012 period, with SMRs of 1.01 (95% CI 0.99, 1.04) during 1998–2007, 1.19 (1.17, 1.21) during 2008–2012 and 1.17 (1.15, 1.19) during 2013–2018 for all-cause mortality; corresponding values for all-cancer mortality were 1.14 (1.09, 1.19), 1.26 (1.22, 1.30) and 1.21 (1.18, 1.24), respectively. SMRs for colorectal, pancreatic, liver and endometrial cancer mortality were consistently high (>1.5) during the whole study period, while there was no evidence of an association of type 2 diabetes with prostate cancer mortality (SMR 0.99; 0.92, 1.05). SMRs for all non-sex-specific cancers were higher in women than men except for liver cancer, for which men had a higher SMR than women during the whole study period.

## Discussion

To a variable extent, we confirmed the previously reported [3, 4] reductions in all-cause mortality in people with type 2 diabetes among all age groups studied; conversely, all-cancer mortality rates declined in the younger age groups (<65 years) but increased in the older age groups, with increasing proportions of cancer deaths out of all-cause deaths in older people. Upward trends in all-cancer mortality rates were observed in both men and women, people of White ethnicity, in both the least and the most deprived quintiles and in people with normal weight and with severe obesity. With higher baseline rates but smaller increases in all-cancer mortality in men than women and in the most deprived group than the least deprived group, we still observed persistent inequalities by gender and deprivation. However, higher rates and increasing trends in current/former smokers than non-smokers led to widening disparities in both all-cause and all-cancer mortality rates by smoking status. Furthermore, there was evidence of constantly increasing trends in pancreatic, liver and lung cancer mortality rates at all ages; colorectal cancer mortality rates at most ages; breast cancer mortality rates at younger ages; and prostate and endometrial cancer mortality rates at older ages. Compared with the general population, people with type 2 diabetes had a more than 1.5-fold increased risk of colorectal, pancreatic, liver and endometrial cancer mortality.

Temporal variations in the definitions and ascertainment of populations and exposures may influence trend estimates in epidemiological studies. Changes in the quality of diabetes recording in the CPRD over time, possibly in relation to the implementation of the UK Quality and Outcomes Framework in 2004 [21], may have resulted in apparent variations in the characteristics of people with type 2 diabetes, with pre-existing complications and a higher mortality risk more commonly found during the initial years of the cohort. At the same time, the more proactive identification of cases of type 2 diabetes earlier in the trajectory of the disease, alongside changes in diagnostic criteria, the increasing incidence of early-onset type 2 diabetes [22] and the availability of newer glucose-lowering treatments with robust cardioprotective effects, may also have contributed to the pattern of a slight increase in all-cause and all-cancer mortality rates followed by declining trends [23, 24]. Furthermore, the reduced risk of fatal cardiovascular events and the resulting prolonged exposure to diabetes increases the likelihood of being diagnosed with conditions other than cardiovascular disease, including cancer; this may partly explain the overall increasing rates and proportions of cancer deaths over time in the older age groups [25]. Lastly, as is evident from improved cancer survival rates [26], early cancer detection and treatment may also have improved over time and this may disproportionally benefit certain subgroups of people with type 2 diabetes.

Our investigation has some strengths and limitations. First, we derived our cohort from electronic health records of primary care patients who were representative of the general population in England. However, these data are not collected for research purposes and the generalisability of our findings is limited by the characteristics of the included individuals and the potential differences in the healthcare system between the UK and other countries. Although we excluded individuals with type 1 diabetes and our clinical codes were reviewed by clinicians practising in England, misclassification was still possible. Second, to our knowledge this is the first study describing cancer-specific mortality trends by sociodemographic characteristics and risk factors in people with type 2 diabetes in England. Despite the large sample size, there were small numbers of deaths in some groups (e.g. across ethnicity), which prevented us carrying out precise and robust assessments of certain trends or investigating ethnic differences more granularly. Third, we used a modelling approach to estimate age-specific mortality rates to control for the impact of age and diabetes duration; indeed, the mean/median age of individuals with type 2 diabetes differs across countries (e.g. median of 58.5 years in a previous Australian study [27] vs 63.8 years in our study) and the overall trends across risk factors might simply reflect differences in the age composition or diabetes duration of the populations studied [28, 29]. Fourth, as our analyses are descriptive, they should not be interpreted as definitively indicating a causal relationship between sociodemographic characteristics or risk factors and cancer. For example, lung cancer mortality rates were higher in the most deprived group than the least deprived group and in smokers than non-smokers but deprivation and smoking status were not mutually adjusted for when estimating rates in these subgroups. In this respect, it is worth noting that removing the causal exposure associated with the higher cancer mortality rate results in a reduction in cancer-specific mortality rate but, at the same time, the risk of competing cause(s) of death may remain the same or even increase, potentially leading to a higher overall risk of death. Whether the magnitude of the effect for the same change in an exposure (e.g. most vs least deprived) differs across competing causes of deaths should be specifically explored in competing risk analyses. Fifth, many contextual factors, such as changes in timings and treatments of diabetes and/or cancer, may contribute to our observed trends but were not accounted for in our analyses. Finally, individuals with missing data on each factor were not included in the corresponding subgroup analysis but our results are unlikely to be strongly biased, given the small number of missing data (<10%) [30].

While declining trends in the rates of all-cause mortality among people with diabetes, mainly because of reduced vascular mortality rates [27, 28, 31, 32], have been consistently reported in the literature [4, 5, 27, 28, 31–38], the evidence is less clear for cancer, making a coherent understanding of the cancer burden in people with diabetes more difficult. Downward trends in cancer mortality rates have been observed in several studies [5, 27, 28, 31, 32, 34] while upward trends have been reported in Sweden and Taiwan [36, 37]; similarly, proportions of cancer deaths have remained stable in the USA [31, 33] but have increased in Australia and the UK [5, 39]. Notably, these studies estimated the overall rates or proportions, while we investigated age-specific rates and proportions of cancer deaths in more detail. In contrast to increasing trends in cancer mortality rates in young adults with type 2 diabetes reported in other countries [28, 29], we observed increasing trends only at older ages, with a parallel reduction in both rates and proportions of cancer deaths at younger ages (<65 years). Our findings also suggest a slightly increased SMR for cancer mortality, which stabilised over time at 1.2, in line with a previous systematic review with trend analysis [40]. While a similar analysis in Australia suggested initial reductions in SMRs for cancer mortality followed by stable trends, the SMRs also stabilised at approximately 1.2 in around 2010 [29, 39]. Taken together, our results confirm that the burden of cancer has increased in individuals with type 2 diabetes in England. At the same time, we found relevant differences across age groups, with such increases occurring mainly in older individuals.

Inequalities in cancer mortality rates by sociodemographic factors were persistent in our cohort. Consistent with previous findings from meta-analyses [12, 41, 42], in our study the SMRs for some cancers were higher in women than men; however, these results should be interpreted alongside the lower baseline cancer mortality rates in women than men. Moreover, in line with cancer mortality data in people with diabetes in the USA [43], we also observed a higher risk of cancer in people of White ethnicity than in people of other ethnicities and a higher risk in the most deprived group than in the least deprived group. However, in contrast to the stable gaps across ethnicity and deprivation observed in the same US study [43], we observed a narrowing then widening but persistent gap across ethnicity and socioeconomic status. These divergent findings may be related to differences between the two countries in social and healthcare systems and their reforms in the last two decades [44, 45] and in the measurement of socioeconomic status and classification of ethnicities. Of note, clinical coding of ethnicities has improved over time in the CPRD [46], which may also have influenced our results.

Few studies have reported cancer mortality rates by risk factors (i.e. smoking and obesity) in people with type 2 diabetes. Our results show that the cancer mortality rates in people with obesity were lower than those in people with normal weight at the start of the observation period, similar to the findings of a previous study carried out in the USA [43]. However, we also found a smaller increase in people with normal weight than in those with severe obesity, leading to similar rates between these two groups during the last years of observation. To our knowledge, this is the first study in people with type 2 diabetes showing constantly higher cancer mortality rates in current and former smokers than non-smokers, in parallel with a steady increase among smokers, leading to widening gaps in cancer mortality rates between smokers and non-smokers.

Our study has important clinical and public health implications. The prevention of cardiovascular disease has been, and is still considered, a priority in people with diabetes. Our results challenge this view by showing that cancer may have overtaken cardiovascular disease as a leading cause of death in people with type 2 diabetes. Cancer prevention strategies therefore deserve at least a similar level of attention as cardiovascular disease prevention [47], particularly in older people and for some cancers such as liver, colorectal and pancreatic cancer. Tailored interventions should also be considered for smokers, who had higher and steadily increasing cancer mortality rates. Early cancer detection through changes to existing screening programmes, or more in-depth investigations for suspected/non-specific symptoms [48], may reduce the number of avoidable cancer deaths in people with type 2 diabetes. From this perspective, our results suggest that it may be helpful to extend breast cancer screening to young women with type 2 diabetes. However, given the high cost and potentially longer exposure to screening procedures, cost-effectiveness analyses are required to define the appropriate time window and identify subgroups who may benefit more. Finally, the number of people with concurrent cancer and type 2 diabetes will be likely to increase in the future, highlighting the importance of improving multidisciplinary clinical management in these patients.

In conclusion, our findings underline the growing cancer burden in people with type 2 diabetes, particularly in older individuals, and highlight the need to prioritise cancer prevention, research and early detection and management in this population, especially for colorectal, pancreatic, liver and endometrial cancer, whose mortality rates were substantially higher in individuals with type 2 diabetes than in the general population. Persistent inequalities in cancer mortality rates by sociodemographic factors and widening disparities by smoking status suggest that tailored cancer prevention and detection strategies are needed. For example, some subgroups such as smokers experienced not only higher mortality rates but also increasing mortality trends during the study period.

## Supplementary information

ESM 1 (PDF 795 kb)

## Data Availability

Data access is through permission from the CPRD only; enquiries should be addressed to enquiries@cprd.com. All clinical code lists and statistical codes are available online (https://github.com/supingling/cancerindiabetes).

## Abbreviations

AAPC: Average annual percentage change
APC: Annual percentage change
CPRD: Clinical Practice Research Datalink
HES: Hospital Episode Statistics
IMD: Index of Multiple Deprivation
ONS: Office for National Statistics
SMR: Standardised mortality ratio
