# Supplementary material for: Inequalities in cancer mortality trends in people with type 2 diabetes: 20 year population-based study in England
Source: Diabetologia. 2023 Jan 24;66(4):657–73. doi: 10.1007/s00125-022-05854-8 (PMC9947024; doi:10.1007/s00125-022-05854-8)
Supplement: Supplementary file 1 — (PDF 795 kb) [file 125_2022_5854_MOESM1_ESM.pdf]

Electronic Supplementary Material

Inequalities in cancer mortality trends in people with type 2 diabetes: 20-year population-based study in England

Suping Ling, Francesco Zaccardi, Eyad Issa, Melanie Davies, Kamlesh Khunti, Karen Brown

Contents

Additional results: All-cause mortality rates 2

ESM Table 1. Trends in all-cause and all cancer mortality rates and proportions of cancer death by age 3

ESM Table 2. Trends in all-cause and all cancer mortality rate and proportions of cancer death by gender 4

ESM Table 3. Trends in all-cause and all cancer mortality rate and proportions of cancer death by ethnicity 5

ESM Table 4. Trends in all-cause and all cancer mortality rate and proportions of cancer death by socio-economic status.... 6

ESM Table 5. Trends in all-cause and all cancer mortality rate and proportions of cancer death by body mass index 7

ESM Table 6. Trends in all-cause and all cancer mortality rate and proportions of cancer death by smoking status 8

ESM Table 7. Annual percentage change and average annual percentage change in cancer-specific mortality rates (four most common cancers) 9

ESM Table 8. Annual percentage change and average annual percentage change in cancer-specific mortality rates (four type 2 diabetes-related cancers) 11

ESM Figure 1. Study participants selection flowchart 13

RECORD checklist 14

Additional results: All-cause mortality rates

Figure 1 shows all-cause mortality rates and corresponding 95% confidence intervals (CIs) from 1998 to 2018 by age (**Figure 1a**), gender (**Figure 1b**), ethnicity (**Figure 1c**), socioeconomic status (**Figure 1d**), BMI groups (**Figure 1e**), and smoking status (**Figure 1f**) with corresponding values in **ESM Tables 1-6**; APCs and AAPCs are presented in **Table 3**.

Overall, mortality rates in individuals with type 2 diabetes declined, with a small increase from 1998 to 2008 but a reduction thereafter, at all ages (**Figure 1**; **ESM Table 1**). For 55-year-old individuals, the mortality rate was 6.5 (95% CI: 5.3, 8.1) per 1000 person-years in 1998, 8.5 (7.9, 9.1) in 2008, and 5.0 (4.3, 5.9) in 2018; the resulting AAPC for the whole study period was -1.1% (-1.4, -0.8), indicating an annual 1.1% reduction from 1998 to 2018 (i.e., 22% reduction in the whole period of 20 years). Corresponding AAPCs for 65-, 75-, and 85-year-old were -1.2% (-1.4, -1.0), -1.8% (-2.1, -1.6), and -1.5% (-1.6, -1.4), respectively (**Table 3**).

Similar trends were observed in men and women. Although all-cause mortality rates were higher in men throughout the study period, there was a greater annual reduction in men [AAPC: -1.9% (95% CI: -2.1, -1.7)] than women [-1.1% (-1.3, -0.9)], leading to a narrowed gap by gender in more recent years (**Figure 1b**; **Table 3**; **ESM Table 2)**.

Trends among people of White ethnicity were slightly different to other ethnicities, with an overall increase in White (0.6%; 0.5, 0.8) but a reduction in other ethnicities (-3.3%; -3.5, -3.1) over the entire study period (**Figure 1c**; **Table 3**; **ESM Table 3**).

When stratified by IMD quintiles, rates in the most deprived group were always higher than in least deprived. Reductions in all-cause mortality rates were higher in the least deprived (-1.3%; -1.8, -0.7) than in the most deprived (-0.6%; -0.9, -0.3), resulting in wider socioeconomic inequalities (**Figure 1d**; **Table 3**; **ESM Table 4)**.

All-cause mortality rates or its trends were not differentiated across BMI groups, with reductions observed in all groups, yet such reduction was smallest in people who were severely obese (-0.6%; -0.7, -0.4) compared to other groups (**Figure 1e**; **Table 3**; **ESM Table 5)**.

Rates were markedly higher in current than former or non-smokers, and gaps were wider in recent years, with an increase in the rates in current smoker (0.3%; 0.2, 0.5) and a reduction in former (-1.9%; -2.0, -1.8) or non-smoker (-2.6%; -2.8, -2.4) (**Figure 1f**; **Table 3**; **ESM Table 6)**.

ESM Table 1. Trends in all-cause and all cancer mortality rates and proportions of cancer death by age

Period	55-year-old			65-year-old		
	All-cause	All cancer	Proportion	All-cause	All cancer	Proportion
1998	6.5 (5.3, 8.1)	2.1 (1.5, 3.1)	32.7 (23.7, 41.7)	16.2 (14.3, 18.2)	5.2 (4.2, 6.4)	32.0 (26.8, 38.0)
1999	6.7 (5.5, 8.1)	2.2 (1.6, 3.0)	32.1 (24.2, 40.1)	16.4 (14.7, 18.2)	5.3 (4.4, 6.4)	32.3 (27.5, 37.7)
2000	6.9 (5.8, 8.2)	2.2 (1.7, 2.9)	31.6 (24.7, 38.6)	16.6 (15.2, 18.3)	5.4 (4.6, 6.4)	32.7 (28.3, 37.4)
2001	7.1 (6.1, 8.2)	2.2 (1.7, 2.8)	31.1 (25.2, 37.0)	16.9 (15.6, 18.3)	5.6 (4.8, 6.5)	33.0 (29.1, 37.0)
2002	7.3 (6.4, 8.2)	2.2 (1.8, 2.7)	30.6 (25.7, 35.6)	17.1 (16.0, 18.3)	5.7 (5.0, 6.5)	33.3 (29.8, 36.6)
2003	7.5 (6.8, 8.3)	2.3 (1.9, 2.7)	30.2 (25.9, 34.3)	17.4 (16.4, 18.4)	5.9 (5.3, 6.5)	33.7 (30.6, 36.5)
2004	7.7 (7.1, 8.4)	2.3 (2.0, 2.6)	29.7 (26.1, 33.3)	17.6 (16.8, 18.5)	6.0 (5.5, 6.6)	34.0 (31.3, 36.5)
2005	7.9 (7.4, 8.5)	2.3 (2.0, 2.6)	29.2 (26.2, 32.2)	17.9 (17.1, 18.7)	6.2 (5.7, 6.7)	34.4 (32.0, 36.7)
2006	8.1 (7.6, 8.7)	2.3 (2.1, 2.6)	28.8 (25.8, 31.6)	18.1 (17.4, 18.9)	6.3 (5.8, 6.8)	34.8 (32.6, 37.1)
2007	8.3 (7.8, 8.9)	2.4 (2.1, 2.7)	28.4 (25.3, 31.4)	18.4 (17.6, 19.1)	6.5 (6.0, 7.0)	35.2 (33.0, 37.7)
2008	8.5 (7.9, 9.1)	2.4 (2.1, 2.7)	28.3 (25.1, 31.4)	18.4 (17.6, 19.2)	6.6 (6.0, 7.1)	35.7 (33.4, 38.4)
2009	8.5 (7.9, 9.1)	2.4 (2.1, 2.8)	28.7 (25.8, 31.8)	18.1 (17.4, 18.9)	6.6 (6.1, 7.1)	36.4 (34.1, 39.1)
2010	8.3 (7.8, 8.8)	2.5 (2.2, 2.8)	29.8 (27.2, 32.8)	17.4 (16.7, 18.0)	6.5 (6.1, 7.0)	37.4 (35.3, 39.8)
2011	7.9 (7.4, 8.5)	2.5 (2.2, 2.8)	31.5 (28.6, 34.7)	16.4 (15.8, 17.0)	6.3 (5.9, 6.8)	38.6 (36.3, 41.0)
2012	7.6 (7.0, 8.2)	2.5 (2.2, 2.9)	33.1 (29.4, 37.1)	15.6 (14.9, 16.3)	6.1 (5.6, 6.7)	39.3 (36.5, 42.1)
2013	7.4 (6.9, 8.0)	2.5 (2.2, 2.8)	33.8 (30.4, 37.3)	15.4 (14.7, 16.0)	5.9 (5.5, 6.4)	38.7 (36.3, 41.4)
2014	7.3 (6.8, 7.8)	2.4 (2.1, 2.7)	33.2 (30.1, 36.7)	15.7 (15.1, 16.3)	5.8 (5.4, 6.3)	37.1 (34.8, 39.5)
2015	7.1 (6.5, 7.7)	2.3 (2.0, 2.6)	32.3 (28.4, 36.0)	15.8 (15.1, 16.5)	5.7 (5.2, 6.2)	35.9 (33.5, 38.8)
2016	6.5 (6.0, 7.0)	2.1 (1.8, 2.4)	31.7 (28.2, 35.0)	15.0 (14.4, 15.6)	5.4 (5.0, 5.9)	36.4 (34.1, 38.8)
2017	5.8 (5.3, 6.3)	1.8 (1.5, 2.1)	31.6 (27.8, 35.6)	13.6 (13.0, 14.2)	5.2 (4.8, 5.6)	38.1 (35.5, 41.1)
2018	5.0 (4.3, 5.9)	1.6 (1.2, 2.1)	31.7 (25.5, 38.4)	12.2 (11.3, 13.1)	4.9 (4.3, 5.7)	40.4 (36.2, 45.2)
**Period**	**75-year-old**			**85-year-old**		
	**All-cause**	**All cancer**	**Proportion**	**All-cause**	**All cancer**	**Proportion**
1998	41.6 (37.2, 46.4)	8.8 (7.0, 11.1)	21.2 (17.4, 25.5)	114.8 (101.0, 130.5)	14.7 (11.2, 19.4)	12.8 (10.0, 16.2)
1999	41.7 (37.8, 46.0)	9.3 (7.6, 11.5)	22.3 (18.7, 26.4)	114.8 (102.4, 128.7)	15.4 (12.0, 19.7)	13.4 (10.8, 16.6)
2000	41.9 (38.4, 45.7)	9.9 (8.2, 11.8)	23.5 (20.0, 27.2)	114.8 (103.9, 126.9)	16.1 (13.0, 20.0)	14.0 (11.5, 17.0)
2001	42.1 (39.0, 45.3)	10.4 (8.9, 12.2)	24.8 (21.4, 28.2)	114.8 (105.3, 125.3)	16.8 (13.9, 20.3)	14.6 (12.4, 17.3)
2002	42.2 (39.6, 45.0)	11.0 (9.6, 12.6)	26.1 (23.0, 29.1)	114.8 (106.6, 123.7)	17.6 (15.0, 20.6)	15.3 (13.3, 17.7)
2003	42.4 (40.1, 44.7)	11.6 (10.4, 13.0)	27.5 (24.7, 30.1)	114.9 (107.9, 122.3)	18.4 (16.1, 21.0)	16.0 (14.1, 18.0)
2004	42.5 (40.6, 44.5)	12.3 (11.2, 13.5)	28.9 (26.4, 31.3)	114.9 (109.0, 121.0)	19.2 (17.2, 21.4)	16.7 (15.0, 18.4)
2005	42.7 (41.0, 44.4)	13.0 (12.0, 14.1)	30.4 (28.2, 32.6)	114.9 (109.9, 120.1)	20.0 (18.3, 22.0)	17.4 (15.9, 18.8)
2006	42.9 (41.3, 44.5)	13.7 (12.7, 14.8)	32.1 (29.8, 34.2)	114.9 (110.4, 119.6)	20.9 (19.2, 22.8)	18.2 (16.7, 19.5)
2007	42.9 (41.3, 44.6)	14.5 (13.4, 15.6)	33.7 (31.6, 35.8)	114.7 (110.1, 119.5)	21.8 (20.0, 23.8)	19.0 (17.5, 20.6)
2008	42.4 (40.7, 44.2)	14.9 (13.7, 16.2)	35.2 (32.9, 37.4)	113.8 (109.1, 118.7)	22.5 (20.6, 24.7)	19.8 (18.2, 21.5)
2009	41.1 (39.6, 42.8)	14.9 (13.8, 16.1)	36.2 (33.8, 38.5)	111.7 (107.2, 116.4)	22.9 (21.0, 25.0)	20.5 (18.8, 22.3)
2010	38.8 (37.5, 40.2)	14.2 (13.2, 15.2)	36.5 (34.3, 38.5)	108.0 (104.3, 112.0)	22.8 (21.1, 24.6)	21.1 (19.5, 22.7)
2011	35.9 (34.7, 37.2)	13.0 (12.1, 14.0)	36.3 (34.0, 38.6)	103.6 (100.0, 107.3)	22.4 (20.7, 24.2)	21.6 (20.0, 23.2)
2012	33.8 (32.4, 35.3)	12.2 (11.2, 13.3)	36.0 (33.6, 38.8)	100.6 (96.5, 104.8)	22.2 (20.3, 24.2)	22.0 (20.4, 23.9)
2013	33.6 (32.3, 34.9)	12.1 (11.2, 13.0)	35.9 (33.6, 38.5)	101.2 (97.5, 105.2)	22.7 (21.0, 24.6)	22.4 (20.9, 24.1)
2014	35.1 (33.8, 36.3)	12.5 (11.7, 13.5)	35.8 (33.6, 38.5)	105.0 (101.6, 108.6)	23.8 (22.1, 25.6)	22.6 (21.2, 24.2)
2015	36.2 (34.7, 37.7)	13.0 (11.9, 14.1)	35.8 (33.4, 38.7)	106.8 (102.7, 111.0)	24.2 (22.3, 26.4)	22.7 (21.1, 24.5)
2016	34.9 (33.6, 36.3)	12.7 (11.8, 13.7)	36.4 (34.2, 38.9)	101.8 (98.2, 105.6)	23.3 (21.6, 25.2)	22.9 (21.4, 24.5)
2017	31.9 (30.6, 33.3)	12.0 (11.1, 13.1)	37.7 (35.0, 40.6)	92.5 (89.0, 96.1)	21.6 (19.9, 23.5)	23.4 (21.7, 25.1)
2018	28.8 (26.9, 30.8)	11.3 (9.9, 12.9)	39.2 (34.6, 43.8)	82.8 (77.9, 88.1)	19.8 (17.3, 22.6)	23.9 (21.2, 26.8)

Proportion (%) and 95% confidence interval of cancer death out of all-cause death.

Rates and 95% confidence interval are presented per 1000 person-years.

ESM Table 2. Trends in all-cause and all cancer mortality rate and proportions of cancer death by gender

Period	Men			Women		
	All-cause	All cancer	Proportion	All-cause	All cancer	Proportion
1998	36.0 (30.6, 42.5)	10.5 (8.1, 13.5)	29.0 (24.1, 35.4)	25.4 (21.0, 30.6)	5.4 (3.9, 7.6)	21.5 (15.8, 27.5)
1999	36.1 (31.1, 41.7)	10.8 (8.6, 13.6)	30.0 (25.4, 35.7)	25.7 (21.7, 30.4)	5.8 (4.3, 7.9)	22.6 (17.2, 28.3)
2000	36.1 (31.7, 41.0)	11.2 (9.2, 13.6)	31.0 (26.7, 36.2)	26.0 (22.5, 30.1)	6.2 (4.7, 8.1)	23.8 (18.7, 28.9)
2001	36.1 (32.3, 40.4)	11.5 (9.7, 13.7)	32.0 (28.0, 36.5)	26.3 (23.2, 29.9)	6.6 (5.2, 8.3)	25.1 (20.2, 29.7)
2002	36.1 (32.8, 39.7)	11.9 (10.3, 13.8)	33.0 (29.4, 37.0)	26.7 (23.9, 29.7)	7.0 (5.8, 8.6)	26.4 (22.1, 30.5)
2003	36.1 (33.3, 39.1)	12.3 (10.8, 14.0)	34.1 (30.8, 37.8)	27.0 (24.7, 29.6)	7.5 (6.4, 8.9)	27.8 (23.7, 31.4)
2004	36.1 (33.7, 38.7)	12.7 (11.4, 14.2)	35.2 (32.4, 38.4)	27.3 (25.3, 29.5)	8.0 (7.0, 9.2)	29.3 (25.6, 32.5)
2005	36.1 (34.1, 38.3)	13.1 (12.0, 14.5)	36.4 (33.6, 39.2)	27.7 (25.9, 29.6)	8.5 (7.6, 9.7)	30.9 (27.6, 33.8)
2006	36.1 (34.2, 38.2)	13.6 (12.4, 14.9)	37.6 (34.8, 40.4)	28.0 (26.3, 29.8)	9.1 (8.1, 10.2)	32.5 (29.4, 35.6)
2007	36.1 (34.1, 38.2)	14.0 (12.7, 15.3)	38.8 (35.7, 41.7)	28.3 (26.5, 30.2)	9.7 (8.6, 10.9)	34.3 (30.9, 37.9)
2008	35.6 (33.5, 37.8)	14.2 (12.8, 15.6)	39.8 (36.6, 42.9)	28.1 (26.3, 30.1)	10.1 (9.0, 11.5)	36.0 (32.4, 40.0)
2009	34.5 (32.5, 36.6)	13.9 (12.7, 15.3)	40.4 (37.4, 43.5)	27.4 (25.7, 29.3)	10.4 (9.2, 11.6)	37.8 (34.2, 41.5)
2010	32.5 (30.9, 34.3)	13.2 (12.1, 14.3)	40.5 (37.9, 43.3)	25.9 (24.5, 27.5)	10.2 (9.2, 11.3)	39.4 (36.0, 42.8)
2011	30.0 (28.5, 31.7)	12.1 (11.1, 13.2)	40.3 (37.7, 43.3)	24.1 (22.7, 25.7)	9.8 (8.8, 11.0)	40.6 (36.7, 44.5)
2012	28.1 (26.4, 29.9)	11.2 (10.1, 12.4)	39.9 (37.1, 43.2)	22.9 (21.2, 24.6)	9.4 (8.3, 10.7)	41.2 (36.6, 45.3)
2013	27.7 (26.1, 29.4)	10.8 (9.9, 11.9)	39.2 (36.5, 42.1)	22.9 (21.5, 24.5)	9.3 (8.3, 10.4)	40.4 (36.5, 44.1)
2014	28.8 (27.3, 30.3)	11.0 (10.1, 12.0)	38.3 (36.0, 41.1)	24.1 (22.7, 25.6)	9.2 (8.3, 10.3)	38.4 (34.9, 42.2)
2015	29.8 (28.0, 31.7)	11.4 (10.3, 12.5)	38.1 (35.4, 41.0)	24.9 (23.2, 26.7)	9.0 (7.9, 10.3)	36.3 (32.4, 40.4)
2016	29.0 (27.4, 30.7)	11.5 (10.5, 12.6)	39.5 (37.1, 42.2)	24.0 (22.5, 25.5)	8.5 (7.6, 9.5)	35.5 (32.1, 39.0)
2017	26.8 (25.3, 28.5)	11.4 (10.3, 12.5)	42.4 (39.5, 45.8)	21.9 (20.4, 23.5)	7.8 (6.8, 8.9)	35.7 (31.4, 39.9)
2018	24.4 (22.1, 27.0)	11.3 (9.6, 13.1)	46.0 (41.0, 51.5)	19.8 (17.6, 22.2)	7.2 (5.8, 8.8)	36.2 (29.9, 42.2)

Proportion (%) and 95% confidence interval of cancer death out of all-cause death.

Rates and 95% confidence interval are presented per 1000 person-years.

ESM Table 3. Trends in all-cause and all cancer mortality rate and proportions of cancer death by ethnicity

Period	White			Others		
	All-cause	All cancer	Proportion	All-cause	All cancer	Proportion
1998	22.2 (19.2, 25.5)	6.6 (5.3, 8.2)	29.6 (25.1, 34.1)	19.0 (9.5, 37.9)	12.3 (3.4, 44.3)	-
1999	23.0 (20.2, 26.1)	7.0 (5.8, 8.5)	30.5 (26.4, 34.5)	18.9 (10.2, 35.1)	11.5 (3.6, 36.1)	-
2000	23.8 (21.3, 26.7)	7.5 (6.3, 8.9)	31.5 (27.6, 35.1)	18.8 (10.8, 32.6)	10.7 (3.8, 29.6)	-
2001	24.7 (22.4, 27.3)	8.0 (6.9, 9.3)	32.4 (28.9, 35.8)	18.7 (11.5, 30.3)	9.9 (4.0, 24.3)	-
2002	25.6 (23.6, 27.9)	8.6 (7.5, 9.8)	33.4 (30.3, 36.4)	18.5 (12.2, 28.3)	9.2 (4.2, 20.0)	-
2003	26.6 (24.8, 28.5)	9.2 (8.2, 10.2)	34.4 (31.6, 37.1)	18.4 (12.8, 26.4)	8.6 (4.4, 16.7)	-
2004	27.6 (26.0, 29.3)	9.8 (8.9, 10.7)	35.5 (32.9, 37.9)	18.3 (13.5, 24.8)	7.9 (4.5, 14.1)	-
2005	28.6 (27.2, 30.1)	10.5 (9.6, 11.3)	36.6 (34.1, 38.8)	18.2 (14.1, 23.5)	7.4 (4.5, 12.1)	-
2006	29.7 (28.4, 31.0)	11.2 (10.4, 12.0)	37.7 (35.2, 39.9)	18.1 (14.5, 22.6)	6.9 (4.4, 10.8)	-
2007	30.7 (29.4, 32.1)	11.9 (11.1, 12.9)	38.8 (36.3, 41.2)	18.0 (14.6, 22.1)	6.4 (4.1, 10.0)	-
2008	31.6 (30.1, 33.2)	12.6 (11.6, 13.6)	39.8 (37.1, 42.4)	17.7 (14.3, 22.0)	5.9 (3.7, 9.5)	-
2009	32.0 (30.5, 33.5)	12.9 (12.0, 14.0)	40.4 (38.0, 43.2)	17.3 (13.8, 21.6)	5.6 (3.4, 9.2)	-
2010	31.4 (30.1, 32.8)	12.8 (11.9, 13.7)	40.6 (38.5, 42.9)	16.3 (13.2, 20.3)	5.3 (3.3, 8.5)	-
2011	30.0 (28.8, 31.2)	12.1 (11.3, 13.0)	40.4 (38.2, 42.9)	14.9 (12.3, 18.0)	5.1 (3.3, 7.8)	-
2012	28.5 (27.1, 30.0)	11.4 (10.6, 12.4)	40.2 (37.6, 43.1)	13.3 (10.8, 16.3)	5.0 (3.2, 7.8)	-
2013	28.1 (26.8, 29.6)	11.2 (10.4, 12.1)	39.7 (37.3, 42.3)	12.4 (9.8, 15.8)	4.9 (2.8, 8.4)	-
2014	29.3 (28.1, 30.6)	11.3 (10.6, 12.2)	38.7 (36.5, 40.9)	12.9 (10.5, 16.0)	4.7 (2.8, 7.7)	-
2015	30.5 (29.0, 32.1)	11.6 (10.7, 12.6)	38.0 (35.5, 40.6)	14.5 (11.7, 17.9)	4.4 (2.6, 7.4)	-
2016	29.9 (28.5, 31.4)	11.5 (10.7, 12.4)	38.5 (36.2, 41.1)	14.6 (11.5, 18.5)	4.5 (2.5, 8.2)	-
2017	27.8 (26.5, 29.2)	11.2 (10.4, 12.2)	40.4 (37.9, 43.0)	12.2 (9.8, 15.3)	5.2 (3.2, 8.5)	-
2018	25.5 (23.5, 27.7)	10.9 (9.6, 12.4)	42.8 (38.7, 47.2)	9.5 (6.5, 14.0)	6.2 (2.8, 13.9)	-

Proportion (%) and 95% confidence interval of cancer death out of all-cause death.

Rates and 95% confidence interval are presented per 1000 person-years.

As the number of deaths among other ethnicities (South Asian, Black and other ethnic groups) that occurred in some years was very small, the proportions of cancer deaths out of all-cause deaths for other ethnicities were not estimated.

ESM Table 4. Trends in all-cause and all cancer mortality rate and proportions of cancer death by socio-economic status

Period	IMD – 1st quintile (Least deprived)			IMD – 2nd quintile			IMD – 3rd quintile		
	All-cause	All cancer	Proportion	All-cause	All cancer	Proportion	All-cause	All cancer	Proportion
1998	21.8 (16.0, 29.7)	5.6 (3.4, 9.2)	25.9 (17.8, 36.4)	29.2 (22.5, 38.0)	7.2 (4.7, 11.1)	24.8 (17.5, 32.7)	31.4 (23.6, 41.7)	9.4 (6.0, 14.8)	29.9 (21.3, 41.4)
1999	22.4 (17.0, 29.5)	6.2 (4.0, 9.5)	27.5 (19.9, 36.8)	29.0 (23.0, 36.7)	7.6 (5.2, 11.1)	26.2 (19.2, 33.3)	31.4 (24.4, 40.4)	9.6 (6.4, 14.3)	30.5 (22.4, 40.8)
2000	23.1 (18.1, 29.4)	6.7 (4.6, 9.9)	29.1 (21.8, 37.7)	28.9 (23.5, 35.5)	8.0 (5.7, 11.1)	27.6 (21.0, 34.3)	31.4 (25.1, 39.2)	9.8 (6.8, 13.9)	31.1 (24.0, 40.2)
2001	23.7 (19.2, 29.3)	7.3 (5.2, 10.2)	30.9 (24.2, 38.5)	28.7 (24.0, 34.3)	8.3 (6.2, 11.1)	29.1 (23.0, 35.4)	31.4 (25.9, 38.0)	10.0 (7.3, 13.6)	31.8 (25.1, 39.9)
2002	24.4 (20.4, 29.3)	8.0 (6.0, 10.6)	32.7 (26.5, 39.8)	28.5 (24.5, 33.2)	8.7 (6.8, 11.2)	30.6 (25.2, 36.6)	31.4 (26.7, 36.9)	10.2 (7.8, 13.3)	32.4 (26.5, 39.3)
2003	25.2 (21.6, 29.3)	8.7 (6.9, 11.1)	34.7 (28.9, 41.4)	28.4 (25.0, 32.3)	9.2 (7.4, 11.3)	32.3 (27.2, 37.7)	31.4 (27.4, 36.0)	10.4 (8.3, 13.0)	33.1 (27.8, 39.1)
2004	25.9 (22.8, 29.4)	9.5 (7.8, 11.7)	36.8 (31.5, 43.5)	28.2 (25.3, 31.4)	9.6 (8.0, 11.5)	34.0 (29.3, 39.0)	31.4 (28.0, 35.2)	10.6 (8.8, 12.8)	33.8 (28.8, 39.5)
2005	26.7 (23.9, 29.7)	10.4 (8.7, 12.4)	39.0 (33.9, 45.3)	28.1 (25.5, 30.8)	10.1 (8.6, 11.8)	35.9 (31.2, 40.7)	31.4 (28.4, 34.6)	10.8 (9.1, 12.8)	34.5 (30.1, 39.4)
2006	27.4 (24.8, 30.3)	11.3 (9.6, 13.4)	41.3 (36.2, 47.5)	27.9 (25.5, 30.5)	10.6 (9.1, 12.3)	37.8 (32.7, 42.9)	31.4 (28.5, 34.5)	11.0 (9.4, 13.0)	35.2 (30.6, 39.9)
2007	28.1 (25.4, 31.2)	12.2 (10.3, 14.6)	43.5 (37.9, 49.6)	27.7 (25.3, 30.4)	11.0 (9.4, 12.9)	39.8 (34.6, 45.2)	31.2 (28.3, 34.5)	11.2 (9.5, 13.3)	36.0 (31.0, 41.3)
2008	28.3 (25.4, 31.6)	12.8 (10.7, 15.3)	45.1 (39.3, 51.7)	27.5 (24.9, 30.3)	11.4 (9.7, 13.4)	41.4 (35.9, 47.4)	30.8 (27.7, 34.1)	11.4 (9.5, 13.5)	36.9 (31.4, 42.5)
2009	27.6 (24.8, 30.7)	12.6 (10.6, 14.9)	45.6 (39.9, 52.1)	27.2 (24.7, 29.9)	11.5 (9.8, 13.5)	42.3 (36.9, 48.1)	29.7 (26.9, 32.9)	11.3 (9.6, 13.4)	38.0 (32.9, 43.3)
2010	25.6 (23.3, 28.2)	11.5 (9.9, 13.4)	44.9 (39.6, 51.0)	26.7 (24.6, 29.1)	11.3 (9.8, 13.0)	42.2 (37.5, 47.3)	28.1 (25.7, 30.6)	11.1 (9.5, 12.9)	39.5 (34.8, 44.0)
2011	22.8 (20.6, 25.2)	10.0 (8.4, 11.9)	44.0 (38.4, 50.4)	26.2 (24.0, 28.5)	10.8 (9.3, 12.4)	41.2 (36.6, 46.2)	26.2 (23.9, 28.8)	10.7 (9.1, 12.6)	40.9 (35.9, 45.6)
2012	20.6 (18.2, 23.2)	8.9 (7.4, 10.8)	43.5 (38.1, 50.6)	25.5 (23.0, 28.2)	10.2 (8.7, 12.0)	40.0 (34.8, 45.9)	25.2 (22.6, 28.1)	10.5 (8.7, 12.5)	41.5 (35.6, 47.1)
2013	20.0 (17.8, 22.4)	8.7 (7.3, 10.2)	43.4 (38.2, 50.0)	24.7 (22.4, 27.3)	9.8 (8.4, 11.4)	39.7 (34.9, 44.9)	26.0 (23.5, 28.7)	10.5 (8.9, 12.3)	40.4 (35.0, 45.3)
2014	21.0 (19.0, 23.2)	8.8 (7.4, 10.4)	41.9 (36.8, 48.5)	24.0 (22.0, 26.1)	9.7 (8.4, 11.1)	40.3 (35.6, 45.4)	28.2 (25.8, 30.8)	10.7 (9.2, 12.5)	38.1 (33.4, 42.4)
2015	22.0 (19.5, 24.7)	8.8 (7.2, 10.6)	39.9 (34.6, 46.9)	23.3 (21.0, 25.9)	9.6 (8.1, 11.4)	41.3 (36.2, 47.1)	29.5 (26.5, 32.7)	10.9 (9.1, 13.0)	36.9 (32.0, 42.2)
2016	21.2 (19.0, 23.7)	8.3 (7.0, 9.9)	39.3 (34.5, 45.1)	22.9 (20.8, 25.2)	9.6 (8.2, 11.2)	42.0 (37.3, 47.1)	27.5 (25.0, 30.2)	10.7 (9.1, 12.5)	38.8 (34.1, 43.9)
2017	19.2 (17.1, 21.5)	7.7 (6.3, 9.3)	40.1 (34.3, 47.0)	22.6 (20.5, 24.9)	9.6 (8.1, 11.3)	42.5 (37.0, 47.4)	23.6 (21.2, 26.3)	10.2 (8.5, 12.2)	43.2 (37.3, 49.5)
2018	17.0 (14.0, 20.5)	7.0 (5.2, 9.6)	41.5 (32.2, 52.3)	22.4 (19.0, 26.3)	9.6 (7.3, 12.5)	42.8 (33.9, 52.2)	19.8 (16.6, 23.7)	9.7 (7.3, 12.9)	49.0 (39.6, 60.7)

Period	IMD – 4th quintile			IMD – 5th quintile (Most deprived)		
	All-cause	All cancer	Proportion	All-cause	All cancer	Proportion
1998	40.0 (30.3, 52.9)	10.1 (6.4, 15.8)	25.2 (17.9, 35.2)	33.6 (25.3, 44.5)	9.8 (6.1, 15.9)	29.2 (19.0, 41.8)
1999	39.6 (30.9, 50.7)	10.4 (6.9, 15.5)	26.1 (19.1, 35.3)	34.6 (26.9, 44.4)	10.1 (6.6, 15.5)	29.3 (19.9, 40.1)
2000	39.2 (31.5, 48.7)	10.6 (7.5, 15.1)	27.1 (20.5, 35.4)	35.6 (28.5, 44.3)	10.4 (7.1, 15.3)	29.3 (20.8, 38.7)
2001	38.8 (32.1, 46.8)	10.9 (8.0, 14.8)	28.1 (21.9, 35.5)	36.6 (30.2, 44.3)	10.7 (7.7, 15.0)	29.4 (21.8, 37.3)
2002	38.4 (32.7, 45.1)	11.2 (8.6, 14.5)	29.1 (23.5, 35.7)	37.7 (32.0, 44.3)	11.1 (8.3, 14.8)	29.4 (22.9, 35.8)
2003	38.0 (33.2, 43.5)	11.5 (9.2, 14.3)	30.1 (25.0, 35.8)	38.8 (33.8, 44.5)	11.4 (8.9, 14.6)	29.5 (23.8, 34.7)
2004	37.6 (33.6, 42.1)	11.7 (9.7, 14.2)	31.2 (26.5, 36.0)	39.9 (35.6, 44.7)	11.8 (9.6, 14.5)	29.5 (24.5, 34.2)
2005	37.2 (33.7, 41.0)	12.0 (10.2, 14.2)	32.4 (28.0, 36.5)	41.0 (37.2, 45.3)	12.1 (10.1, 14.5)	29.6 (25.1, 33.9)
2006	36.8 (33.6, 40.4)	12.3 (10.5, 14.5)	33.5 (29.2, 37.9)	42.2 (38.5, 46.3)	12.5 (10.6, 14.8)	29.6 (25.3, 33.7)
2007	36.4 (33.0, 40.0)	12.6 (10.7, 14.8)	34.7 (29.9, 39.6)	43.3 (39.3, 47.6)	12.9 (10.9, 15.3)	29.8 (25.4, 34.2)
2008	35.6 (32.2, 39.4)	12.7 (10.7, 15.1)	35.7 (30.8, 41.2)	43.5 (39.4, 48.0)	13.2 (11.1, 15.8)	30.5 (25.8, 35.3)
2009	34.3 (31.1, 37.9)	12.5 (10.7, 14.7)	36.5 (31.7, 41.9)	42.3 (38.4, 46.5)	13.5 (11.3, 16.1)	32.0 (27.1, 37.3)
2010	32.5 (29.8, 35.4)	12.0 (10.4, 13.8)	36.8 (32.6, 41.3)	39.2 (36.0, 42.6)	13.6 (11.7, 15.9)	34.8 (30.5, 40.0)
2011	30.3 (27.7, 33.2)	11.2 (9.6, 13.0)	36.8 (32.3, 41.3)	35.4 (32.3, 38.7)	13.6 (11.7, 15.8)	38.4 (33.6, 43.6)
2012	28.9 (25.9, 32.1)	10.5 (8.8, 12.5)	36.5 (31.6, 41.6)	32.8 (29.5, 36.4)	13.3 (11.2, 15.9)	40.7 (35.2, 47.0)
2013	28.8 (26.1, 31.8)	10.3 (8.8, 12.0)	35.8 (31.5, 40.3)	32.9 (29.9, 36.1)	12.9 (10.9, 15.2)	39.3 (34.1, 45.0)
2014	30.0 (27.5, 32.8)	10.5 (9.0, 12.2)	34.9 (30.4, 39.1)	35.3 (32.3, 38.5)	12.4 (10.6, 14.4)	35.1 (30.9, 40.1)
2015	30.9 (27.8, 34.3)	10.7 (9.0, 12.7)	34.6 (29.7, 39.2)	37.2 (33.6, 41.2)	12.0 (10.0, 14.4)	32.3 (28.2, 37.4)
2016	30.0 (27.2, 33.0)	10.7 (9.1, 12.5)	35.7 (31.1, 40.9)	36.1 (33.0, 39.6)	11.9 (10.1, 14.0)	32.9 (28.6, 37.7)
2017	27.7 (25.0, 30.7)	10.5 (8.9, 12.5)	38.0 (32.8, 43.5)	33.2 (30.0, 36.7)	12.0 (10.1, 14.2)	36.1 (30.9, 41.3)
2018	25.3 (21.3, 30.0)	10.4 (7.9, 13.6)	41.0 (32.9, 50.3)	30.0 (25.5, 35.3)	12.1 (9.2, 15.9)	40.4 (31.4, 49.9)

Proportion (%) and 95% confidence interval of cancer death out of all-cause death. Rates and 95% confidence interval are presented per 1000 person-years. IMD: index of multiple deprivation 2010.

ESM Table 5. Trends in all-cause and all cancer mortality rate and proportions of cancer death by body mass index

Period	Normal weight (BMI: 18.5-24.9 kg/m^2)			Overweight (BMI: 25-29.9 kg/m^2)		
	**All-cause**	**All cancer**	**Proportion**	**All-cause**	**All cancer**	**Proportion**
1998	32.2 (24.4, 42.5)	9.8 (5.7, 16.9)	30.6 (19.0, 49.2)	30.7 (24.8, 38.0)	6.7 (4.6, 9.8)	22.0 (16.3, 29.0)
1999	32.5 (25.4, 41.5)	10.0 (6.2, 16.2)	30.9 (20.3, 47.5)	30.4 (25.1, 36.7)	7.1 (5.1, 10.0)	23.4 (18.0, 30.0)
2000	32.8 (26.5, 40.6)	10.2 (6.8, 15.5)	31.3 (21.7, 45.3)	30.0 (25.4, 35.5)	7.5 (5.6, 10.1)	25.0 (19.8, 31.2)
2001	33.1 (27.5, 39.7)	10.5 (7.3, 14.9)	31.6 (23.1, 43.2)	29.7 (25.7, 34.4)	7.9 (6.1, 10.3)	26.7 (21.8, 32.2)
2002	33.4 (28.6, 39.0)	10.7 (7.9, 14.4)	32.0 (24.3, 41.6)	29.4 (26.0, 33.3)	8.4 (6.7, 10.4)	28.5 (24.0, 33.5)
2003	33.7 (29.6, 38.4)	10.9 (8.5, 14.0)	32.3 (25.5, 40.6)	29.1 (26.2, 32.3)	8.9 (7.4, 10.7)	30.4 (26.2, 35.1)
2004	34.0 (30.5, 38.0)	11.1 (8.9, 13.9)	32.7 (26.6, 39.5)	28.8 (26.3, 31.4)	9.3 (8.0, 10.9)	32.5 (28.8, 36.7)
2005	34.3 (31.1, 38.0)	11.4 (9.2, 14.0)	33.1 (27.4, 39.4)	28.4 (26.3, 30.7)	9.9 (8.6, 11.3)	34.7 (31.0, 38.6)
2006	34.6 (31.3, 38.3)	11.6 (9.3, 14.4)	33.4 (27.8, 40.2)	28.1 (26.2, 30.2)	10.4 (9.2, 11.8)	37.0 (33.0, 41.2)
2007	34.6 (31.1, 38.6)	11.7 (9.3, 14.7)	33.7 (27.9, 41.1)	27.8 (25.8, 29.9)	11.0 (9.6, 12.5)	39.4 (35.0, 44.0)
2008	34.2 (30.7, 38.1)	11.6 (9.3, 14.5)	33.9 (28.0, 41.8)	27.4 (25.3, 29.6)	11.4 (9.9, 13.1)	41.6 (37.0, 46.7)
2009	33.0 (29.9, 36.5)	11.3 (9.2, 13.8)	34.1 (28.8, 41.4)	26.8 (24.8, 29.0)	11.5 (10.1, 13.2)	43.0 (38.4, 48.1)
2010	31.2 (28.4, 34.3)	10.8 (8.8, 13.3)	34.6 (29.1, 41.6)	26.0 (24.2, 27.8)	11.2 (10.0, 12.7)	43.3 (39.2, 47.5)
2011	29.5 (26.3, 33.0)	10.5 (8.3, 13.3)	35.5 (29.0, 44.1)	25.0 (23.3, 26.8)	10.7 (9.4, 12.1)	42.6 (38.1, 47.3)
2012	28.6 (25.5, 32.2)	10.6 (8.4, 13.2)	36.9 (30.2, 44.4)	24.3 (22.4, 26.4)	10.1 (8.7, 11.7)	41.6 (36.6, 47.1)
2013	29.0 (26.3, 32.0)	11.1 (9.1, 13.5)	38.2 (32.4, 44.8)	24.5 (22.6, 26.6)	9.9 (8.7, 11.3)	40.4 (35.9, 44.9)
2014	29.8 (26.7, 33.2)	11.7 (9.3, 14.6)	39.3 (32.4, 48.0)	25.5 (23.8, 27.4)	10.0 (8.8, 11.3)	39.0 (35.0, 43.3)
2015	29.8 (26.5, 33.4)	12.0 (9.6, 15.1)	40.3 (33.1, 49.3)	25.8 (23.7, 28.1)	9.9 (8.5, 11.5)	38.3 (33.4, 43.1)
2016	28.6 (25.9, 31.7)	11.9 (9.7, 14.6)	41.6 (35.1, 50.0)	23.8 (22.0, 25.8)	9.4 (8.2, 10.7)	39.4 (34.8, 43.8)
2017	27.0 (23.8, 30.7)	11.7 (9.0, 15.2)	43.2 (34.1, 54.0)	20.3 (18.7, 22.1)	8.6 (7.4, 10.0)	42.4 (37.2, 47.2)
2018	25.4 (21.0, 30.9)	11.4 (7.7, 16.9)	44.9 (32.0, 61.4)	16.9 (14.6, 19.6)	7.9 (6.2, 10.1)	46.4 (38.2, 55.8)

Period	Obese (BMI: 30-34.9 kg/m^2)			Severely obese (BMI: ≥35 kg/m^2)		
	**All-cause**	**All cancer**	**Proportion**	**All-cause**	**All cancer**	**Proportion**
1998	35.8 (27.6, 46.6)	9.4 (6.3, 14.1)	26.2 (19.2, 35.0)	31.9 (21.9, 46.4)	3.6 (1.5, 8.4)	11.2 (4.8, 23.2)
1999	35.3 (27.9, 44.7)	9.7 (6.7, 13.9)	27.4 (20.6, 35.4)	32.2 (23.0, 45.2)	4.0 (1.8, 8.7)	12.4 (5.7, 23.8)
2000	34.8 (28.2, 42.9)	10.0 (7.2, 13.8)	28.7 (22.2, 35.9)	32.6 (24.1, 44.1)	4.5 (2.2, 9.0)	13.8 (6.9, 24.5)
2001	34.3 (28.6, 41.2)	10.3 (7.8, 13.6)	30.0 (23.9, 36.5)	33.0 (25.2, 43.1)	5.1 (2.7, 9.3)	15.3 (8.3, 25.4)
2002	33.8 (28.9, 39.6)	10.6 (8.3, 13.5)	31.3 (25.7, 37.2)	33.3 (26.4, 42.1)	5.7 (3.3, 9.7)	17.1 (9.9, 26.3)
2003	33.4 (29.1, 38.2)	10.9 (8.9, 13.4)	32.7 (27.6, 38.0)	33.7 (27.6, 41.1)	6.4 (4.0, 10.1)	18.9 (11.9, 27.3)
2004	32.9 (29.3, 36.8)	11.2 (9.4, 13.4)	34.2 (29.7, 39.0)	34.1 (28.8, 40.3)	7.2 (4.9, 10.5)	21.1 (14.1, 28.7)
2005	32.4 (29.4, 35.7)	11.6 (9.9, 13.5)	35.7 (31.7, 40.2)	34.4 (29.9, 39.7)	8.1 (5.9, 11.1)	23.4 (16.9, 30.0)
2006	31.9 (29.3, 34.8)	11.9 (10.4, 13.7)	37.4 (33.5, 41.5)	34.8 (30.9, 39.2)	9.1 (7.0, 11.8)	26.0 (19.8, 31.9)
2007	31.5 (29.0, 34.2)	12.3 (10.7, 14.1)	39.0 (35.2, 42.9)	35.2 (31.6, 39.1)	10.2 (8.1, 12.7)	28.9 (23.1, 34.6)
2008	31.0 (28.3, 33.8)	12.5 (10.9, 14.4)	40.5 (36.6, 44.8)	35.5 (32.0, 39.5)	11.4 (9.2, 14.2)	32.1 (26.1, 38.3)
2009	30.1 (27.5, 33.1)	12.5 (10.8, 14.4)	41.5 (37.5, 46.0)	35.7 (31.9, 39.9)	12.6 (10.0, 15.8)	35.2 (27.9, 42.1)
2010	28.9 (26.5, 31.5)	12.0 (10.5, 13.7)	41.6 (38.0, 45.8)	35.4 (31.7, 39.6)	13.3 (10.6, 16.7)	37.4 (30.1, 44.6)
2011	27.1 (25.1, 29.3)	11.0 (9.8, 12.5)	40.8 (37.2, 44.9)	34.5 (31.3, 38.1)	13.1 (10.7, 16.0)	37.9 (31.3, 44.6)
2012	25.2 (23.0, 27.6)	10.1 (8.7, 11.6)	39.9 (35.5, 44.8)	33.0 (30.0, 36.2)	11.9 (9.7, 14.6)	36.2 (30.2, 42.3)
2013	24.0 (21.7, 26.5)	9.5 (8.2, 11.1)	39.8 (35.4, 44.8)	31.5 (28.1, 35.4)	10.7 (8.3, 13.7)	33.9 (27.0, 41.3)
2014	23.9 (22.0, 26.1)	9.7 (8.6, 11.0)	40.6 (36.4, 44.6)	31.2 (28.0, 34.7)	10.2 (8.2, 12.8)	32.8 (27.0, 39.3)
2015	24.5 (22.3, 27.0)	10.1 (8.7, 11.7)	41.3 (36.4, 46.0)	32.0 (29.0, 35.3)	10.6 (8.5, 13.2)	33.1 (27.1, 40.2)
2016	24.6 (22.3, 27.1)	10.2 (8.9, 11.8)	41.7 (37.2, 47.0)	32.2 (28.7, 36.0)	11.1 (8.8, 14.1)	34.7 (28.4, 42.2)
2017	23.8 (21.7, 26.1)	10.0 (8.7, 11.5)	42.0 (37.7, 46.5)	30.7 (27.8, 33.8)	11.5 (9.4, 14.1)	37.6 (31.3, 43.5)
2018	22.7 (19.4, 26.6)	9.6 (7.7, 12.1)	42.4 (35.2, 50.0)	28.6 (24.2, 33.8)	11.8 (8.4, 16.6)	41.4 (29.7, 53.7)

Proportion (%) and 95% confidence interval of cancer death out of all-cause death.

Rates and 95% confidence interval are presented per 1000 person-years.

BMI: body mass index. Subjects with underweight (BMI: <18.5 kg/m^2) were not included in this analysis.

ESM Table 6. Trends in all-cause and all cancer mortality rate and proportions of cancer death by smoking status

Period	Current smoker			Non-smoker			Ex-smoker		
	**All-cause**	**All cancer**	**Proportion**	**All-cause**	**All cancer**	**Proportion**	**All-cause**	**All cancer**	**Proportion**
1998	40.0 (29.5, 54.3)	8.8 (4.8, 16.1)	22.0 (12.9, 33.6)	24.9 (20.6, 30.2)	8.1 (5.8, 11.3)	32.4 (24.3, 41.0)	36.3 (28.9, 45.5)	8.5 (5.8, 12.6)	23.5 (16.7, 31.6)
1999	41.0 (31.3, 53.7)	9.4 (5.5, 16.1)	22.8 (14.2, 33.5)	24.8 (20.9, 29.4)	8.2 (6.1, 11.1)	33.1 (25.6, 40.6)	36.0 (29.4, 44.1)	8.8 (6.2, 12.5)	24.6 (18.1, 32.2)
2000	42.0 (33.2, 53.3)	10.0 (6.2, 16.0)	23.7 (15.7, 33.3)	24.6 (21.2, 28.6)	8.4 (6.4, 10.9)	33.9 (26.9, 40.4)	35.7 (29.8, 42.7)	9.2 (6.7, 12.5)	25.8 (19.8, 32.6)
2001	43.1 (35.1, 52.8)	10.6 (7.1, 16.0)	24.7 (17.1, 33.1)	24.5 (21.5, 27.9)	8.5 (6.8, 10.7)	34.7 (28.4, 40.4)	35.4 (30.2, 41.4)	9.6 (7.3, 12.5)	27.0 (21.4, 33.3)
2002	44.1 (37.1, 52.5)	11.3 (8.0, 16.0)	25.6 (18.8, 33.1)	24.4 (21.8, 27.2)	8.6 (7.1, 10.5)	35.5 (29.8, 40.5)	35.1 (30.7, 40.2)	9.9 (7.9, 12.5)	28.3 (23.3, 34.1)
2003	45.2 (39.1, 52.3)	12.1 (9.0, 16.2)	26.7 (20.6, 33.1)	24.2 (22.1, 26.6)	8.8 (7.5, 10.4)	36.3 (31.2, 40.6)	34.8 (31.0, 39.1)	10.3 (8.5, 12.6)	29.6 (25.0, 34.9)
2004	46.3 (41.0, 52.4)	12.8 (10.1, 16.4)	27.7 (22.3, 33.3)	24.1 (22.3, 26.0)	8.9 (7.8, 10.3)	37.1 (32.7, 41.0)	34.5 (31.4, 38.1)	10.7 (9.1, 12.7)	31.0 (27.1, 35.7)
2005	47.5 (42.7, 52.8)	13.7 (11.1, 16.9)	28.8 (24.4, 34.0)	24.0 (22.4, 25.6)	9.1 (8.0, 10.3)	38.0 (34.0, 41.9)	34.3 (31.6, 37.2)	11.1 (9.7, 12.9)	32.5 (28.7, 37.0)
2006	48.7 (44.0, 53.8)	14.6 (12.0, 17.7)	30.0 (25.4, 35.1)	23.8 (22.3, 25.4)	9.3 (8.2, 10.5)	38.8 (34.7, 43.0)	34.0 (31.6, 36.6)	11.6 (10.2, 13.2)	34.1 (30.3, 38.1)
2007	49.7 (44.7, 55.3)	15.5 (12.7, 19.0)	31.2 (25.9, 37.0)	23.6 (22.1, 25.3)	9.4 (8.2, 10.7)	39.7 (35.5, 44.1)	33.7 (31.4, 36.2)	12.0 (10.6, 13.6)	35.7 (32.0, 39.6)
2008	50.3 (45.0, 56.2)	16.4 (13.2, 20.2)	32.5 (26.7, 39.4)	23.2 (21.6, 24.9)	9.4 (8.2, 10.7)	40.4 (35.9, 45.1)	33.3 (30.9, 36.0)	12.4 (10.9, 14.2)	37.2 (33.5, 41.4)
2009	50.2 (45.2, 55.8)	17.1 (14.0, 21.0)	34.1 (28.4, 40.8)	22.4 (20.9, 24.0)	9.2 (8.1, 10.5)	41.2 (36.9, 45.6)	32.7 (30.2, 35.4)	12.5 (10.9, 14.3)	38.3 (34.2, 42.7)
2010	49.1 (44.8, 53.8)	17.6 (14.7, 21.0)	35.9 (31.1, 41.3)	21.1 (19.8, 22.4)	8.8 (7.9, 9.9)	41.8 (37.9, 45.9)	31.6 (29.4, 34.0)	12.2 (10.8, 13.8)	38.5 (34.7, 42.6)
2011	47.3 (43.0, 52.1)	17.8 (14.8, 21.3)	37.6 (32.6, 43.4)	19.6 (18.3, 20.9)	8.3 (7.3, 9.4)	42.4 (37.8, 47.1)	30.1 (28.2, 32.2)	11.4 (10.2, 12.8)	38.0 (34.4, 41.6)
2012	45.8 (41.0, 51.2)	17.7 (14.4, 21.9)	38.7 (32.6, 46.2)	18.4 (17.0, 19.9)	7.8 (6.8, 9.0)	42.3 (36.8, 47.5)	28.6 (26.5, 30.9)	10.7 (9.3, 12.2)	37.2 (33.3, 41.6)
2013	45.3 (41.0, 50.1)	17.5 (14.4, 21.1)	38.5 (32.5, 44.8)	18.1 (16.8, 19.4)	7.4 (6.5, 8.4)	40.9 (36.5, 45.3)	28.0 (25.8, 30.5)	10.3 (9.0, 11.8)	36.9 (33.0, 41.1)
2014	45.8 (41.8, 50.2)	17.1 (14.4, 20.4)	37.4 (31.8, 43.4)	18.5 (17.3, 19.7)	7.1 (6.2, 8.0)	38.4 (34.3, 43.1)	28.8 (26.9, 30.8)	10.6 (9.5, 11.9)	36.9 (33.3, 40.5)
2015	46.2 (41.4, 51.4)	16.9 (13.8, 20.8)	36.7 (30.7, 44.2)	18.6 (17.2, 20.2)	6.8 (5.9, 7.9)	36.7 (32.3, 41.8)	29.7 (27.4, 32.1)	11.0 (9.6, 12.6)	37.0 (33.2, 41.6)
2016	45.6 (41.4, 50.3)	16.9 (14.1, 20.4)	37.2 (31.6, 43.6)	17.6 (16.4, 18.9)	6.6 (5.8, 7.5)	37.3 (33.4, 41.8)	29.1 (26.9, 31.4)	10.9 (9.6, 12.4)	37.5 (33.9, 41.7)
2017	44.3 (40.1, 49.0)	17.1 (14.2, 20.7)	38.6 (31.9, 44.1)	15.9 (14.7, 17.2)	6.3 (5.5, 7.3)	39.8 (35.3, 44.5)	27.0 (25.0, 29.1)	10.4 (9.1, 11.9)	38.6 (35.0, 42.5)
2018	42.9 (36.5, 50.5)	17.4 (12.8, 23.5)	40.5 (31.0, 51.1)	14.1 (12.4, 16.0)	6.1 (4.8, 7.7)	43.1 (35.1, 52.0)	24.6 (21.6, 28.1)	9.9 (7.9, 12.3)	40.0 (34.0, 47.0)

Proportion (%) and 95% confidence interval of cancer death out of all-cause death.

Rates and 95% confidence interval are presented per 1000 person-years.

ESM Table 7. Annual percentage change and average annual percentage change in cancer-specific mortality rates (four most common cancers)

	Period 1	*APC, %*	*Period 2*	*APC, %*	*Period 3*	*APC, %*	*Period 4*	*APC, %*	*AAPC for whole period, %*
Breast									
**Age, years**									
55	1998-2006	12.7 (11.6, 13.8)	2006-2009	8.7 (6.3, 11.1)	2009-2015	-3.1 (-3.5, -2.7)	2015-2018	-6.7 (-8.4, -5.0)	4.1 (3.6, 4.7)
65	1998-2008	9.5 (8.2, 10.7)	2008-2015	-5.9 (-6.8, -4.9)	2015-2018	-15.3 (-22.2, -7.9)	–	–	-0.1 (-1.4, 1.2)
75	1998-2008	3.6 (3.4, 3.8)	2008-2012	-8.3 (-8.9, -7.6)	2012-2015	9.9 (8.4, 11.5)	2015-2018	-12.7 (-13.6, -11.7)	-0.6 (-0.9, -0.3)
85	1998-2008	-0.2 (-0.3, -0.2)	2008-2012	2.7 (2.6, 2.8)	2012-2015	-7.2 (-7.4, -6.9)	2015-2018	1.4 (1.2, 1.6)	-0.5 (-0.5, -0.4)
**Ethnicity**									
White	1998-2009	8.6 (8.2, 9.0)	2009-2012	-10.7 (-12.5, -8.8)	2012-2015	1.8 (-0.8, 4.4)	2015-2018	-15.9 (-18.1, -13.7)	0.5 (-0.1, 1.1)
Others	1998-2008	-33.1 (-34.3, -32.0)	2008-2014	55.7 (8.6, 123.1)	2014-2018	-30.3 (-43.1, -14.6)	–	–	-13.1 (-21.8, -3.5)
**Deprivation**									
Least deprived	1998-2009	5.5 (5.0, 6.0)	2009-2012	-23.6 (-26.7, -20.3)	2012-2015	47.5 (40.7, 54.6)	2015-2018	-32.4 (-35.7, -29.0)	-1.1 (-2.2, 0.0)
Most deprived	1998-2008	-11.6 (-12.2, -11.0)	2008-2012	38.2 (33.8, 42.8)	2012-2016	-27.8 (-30.2, -25.3)	2016-2018	10.4 (-4.4, 27.5)	-5.1 (-6.6, -3.6)
**BMI group, kg/m^2**									
18.5-24.9	1998-2008	1.6 (1.4, 1.7)	2008-2011	-10.4 (-11.4, -9.5)	2011-2015	1.8 (1.1, 2.5)	2015-2018	-27.6 (-29.1, -26.0)	-5.2 (-5.5, -4.9)
25.0-29.9	1998-2006	7.8 (6.0, 9.6)	2006-2009	4.1 (-0.8, 9.3)	2009-2012	-12.1 (-16.7, -7.3)	2012-2018	-2.7 (-4.4, -1.0)	0.8 (-0.4, 2.1)
30.0-34.9	1998-2009	21.7 (20.8, 22.7)	2009-2012	-21.9 (-24.0, -19.7)	2012-2015	16.5 (12.8, 20.4)	2015-2018	-30.6 (-33.4, -27.7)	4.0 (3.1, 4.9)
≥35.0	1998-2007	6.3 (5.7, 7.0)	2007-2010	1.5 (-0.9, 4.0)	2010-2015	-10.5 (-11.3, -9.6)	2015-2018	-28.9 (-33.9, -23.6)	-4.8 (-5.8, -3.8)
**Smoking status**									
Current smoker	1998-2008	1.8 (1.4, 2.1)	2008-2014	-6.8 (-7.4, -6.2)	2014-2018	-22.1 (-24.6, -19.5)	–	–	-6.1 (-6.6, -5.5)
Ex-smoker	1998-2009	11.0 (10.2, 11.8)	2009-2012	-25.2 (-28.5, -21.7)	2012-2015	26.3 (18.9, 34.2)	2015-2018	-26.4 (-30.9, -21.7)	0.3 (-1.0, 1.6)
Non-smoker	1998-2008	7.6 (7.4, 7.8)	2008-2011	-1.6 (-2.3, -0.9)	2011-2018	-10.7 (-10.9, -10.5)	–	–	-0.5 (-0.7, -0.4)
Prostate									
**Age, years**									
55	1998-2012	-5.0 (-5.1, -4.9)	2012-2016	19.3 (18.5, 20.2)	2016-2018	-26.5 (-28.4, -24.6)	–	–	-3.1 (-3.3, -2.8)
65	1998-2012	-1.4 (-1.5, -1.4)	2012-2016	13.1 (12.6, 13.6)	2016-2018	-23.3 (-24.6, -22.0)	–	–	-1.2 (-1.4, -1.0)
75	1998-2011	1.6 (1.5, 1.7)	2011-2016	5.0 (4.7, 5.4)	2016-2018	-14.1 (-15.6, -12.4)	–	–	0.8 (0.6, 1.0)
85	1998-2009	8.1 (7.9, 8.2)	2009-2013	2.9 (2.5, 3.2)	2013-2016	9.2 (8.7, 9.8)	2016-2018	-6.8 (-7.4, -6.2)	5.6 (5.5, 5.7)
**Ethnicity**									
White	1998-2012	3.6 (3.6, 3.6)	2012-2016	10.0 (9.9, 10.2)	2016-2018	-17.4 (-17.7, -17.0)	–	–	2.5 (2.5, 2.6)
Others	1998-2008	42.4 (15.9, 75.0)	2008-2018	-15.2 (-21.0, -8.9)	–	–	–	–	9.9 (-0.6, 21.6)
**Deprivation**									
Least deprived	1998-2008	-7.8 (-8.1, -7.4)	2008-2014	5.6 (4.6, 6.5)	2014-2018	-20.3 (-22.7, -17.9)	–	–	-6.7 (-7.3, -6.1)
Most deprived	1998-2009	7.2 (6.6, 7.8)	2009-2012	-27.9 (-31.4, -24.3)	2012-2016	42.4 (37.9, 47.1)	2016-2018	-38.2 (-43.1, -32.8)	1.2 (0.0, 2.4)
**BMI group, kg/m^2**									
18.5-24.9	1998-2012	7.7 (7.5, 8.0)	2012-2015	25.2 (23.7, 26.7)	2015-2018	-26.1 (-27.6, -24.5)	–	–	4.1 (3.8, 4.5)
25.0-29.9	1998-2008	-9.6 (-9.7, -9.5)	2008-2011	-0.9 (-2.7, 1.0)	2011-2015	13.3 (12.6, 14.1)	2015-2018	-10.4 (-11.2, -9.5)	-4.2 (-4.5, -3.9)
30.0-34.9	1998-2010	2.3 (2.2, 2.4)	2010-2013	-6.0 (-6.9, -5.1)	2013-2016	0.7 (-0.5, 1.9)	2016-2018	-4.0 (-5.6, -2.3)	0.1 (-0.1, 0.4)
≥35.0	1998-2015	24.2 (22.2, 26.2)	2015-2018	-25.3 (-32.6, -17.3)	–	–	–	–	15.1 (12.9, 17.3)
**Smoking status**									
Current smoker	1998-2009	3.7 (3.5, 3.9)	2009-2013	15.9 (15.5, 16.4)	2013-2016	-5.3 (-5.8, -4.8)	2016-2018	-11.4 (-12.4, -10.4)	3.0 (2.8, 3.1)
Ex-smoker	1998-2008	8.5 (8.3, 8.8)	2008-2011	5.9 (5.0, 6.8)	2011-2016	-2.1 (-2.3, -1.8)	2016-2018	-6.8 (-8.1, -5.4)	3.8 (3.6, 4.0)
Non-smoker	1998-2012	-6.1 (-6.7, -5.5)	2012-2016	29.0 (22.0, 36.5)	2016-2018	-37.8 (-50.0, -22.6)	–	–	-4.0 (-6.1, -1.8)
Lung									
**Age, years**									
55	1998-2010	8.5 (8.4, 8.7)	2010-2013	0.6 (-0.2, 1.3)	2013-2018	-16.9 (-17.2, -16.5)	–	–	0.4 (0.2, 0.5)
65	1998-2009	3.5 (3.5, 3.6)	2009-2012	1.2 (1.0, 1.4)	2012-2015	-8.6 (-8.9, -8.4)	2015-2018	-0.4 (-0.6, -0.2)	0.7 (0.6, 0.7)
75	1998-2009	8.6 (8.4, 8.9)	2009-2012	-3.8 (-4.8, -2.8)	2012-2015	2.0 (0.9, 3.2)	2015-2018	-9.8 (-10.7, -8.9)	2.8 (2.5, 3.0)
85	1998-2009	5.7 (5.5, 5.9)	2009-2012	-3.9 (-4.9, -2.8)	2012-2015	7.5 (6.4, 8.6)	2015-2018	-14.0 (-14.7, -13.2)	1.3 (1.1, 1.6)
**Gender**									
Men	1998-2006	3.6 (3.5, 3.7)	2006-2009	2.3 (2.0, 2.6)	2009-2013	-4.2 (-4.3, -4.1)	2013-2018	-0.2 (-0.3, -0.1)	0.9 (0.8, 0.9)

	Period 1	APC, %	Period 2	APC, %	Period 3	APC, %	Period 4	APC, %	AAPC for whole period, %
Women	1998-2009	8.9 (8.9, 9.0)	2009-2012	2.0 (1.6, 2.3)	2012-2015	-5.0 (-5.3, -4.6)	2015-2018	-0.4 (-0.6, -0.1)	4.3 (4.2, 4.4)
**Ethnicity**									
White	1998-2007	7.3 (7.2, 7.4)	2007-2010	5.0 (4.6, 5.5)	2010-2015	-2.3 (-2.4, -2.2)	2015-2018	0.7 (0.4, 0.9)	3.5 (3.4, 3.6)
Others	1998-2010	-17.7 (-17.8, -17.7)	2010-2013	-6.1 (-9.4, -2.7)	2013-2016	28.8 (25.2, 32.6)	2016-2018	-6.6 (-9.1, -4.1)	-9.1 (-9.7, -8.5)
**Deprivation**									
Least deprived	1998-2008	10.7 (10.5, 11.0)	2008-2011	5.7 (5.0, 6.5)	2011-2015	-17.7 (-18.2, -17.3)	2015-2018	-0.7 (-1.7, 0.4)	1.9 (1.7, 2.2)
Most deprived	1998-2008	7.7 (7.6, 7.9)	2008-2012	9.8 (9.6, 10.0)	2012-2016	-5.9 (-6.1, -5.7)	2016-2018	2.7 (2.1, 3.2)	4.8 (4.7, 4.9)
**BMI group, kg/m^2**									
18.5-24.9	1998-2008	11.1 (10.7, 11.4)	2008-2012	-8.2 (-8.8, -7.5)	2012-2015	10.1 (8.6, 11.6)	2015-2018	-13.3 (-14.3, -12.3)	2.9 (2.6, 3.2)
25.0-29.9	1998-2009	1.8 (1.7, 1.8)	2009-2012	-0.5 (-0.7, -0.3)	2012-2018	-5.0 (-5.0, -4.9)	–	–	-0.6 (-0.7, -0.6)
30.0-34.9	1998-2008	2.1 (1.7, 2.5)	2008-2015	5.3 (5.0, 5.6)	2015-2018	-17.7 (-19.0, -16.4)	–	–	-0.1 (-0.4, 0.2)
≥35.0	1998-2009	33.5 (24.7, 43.0)	2009-2013	-17.6 (-24.2, -10.3)	2013-2018	20.2 (16.3, 24.2)	–	–	18.1 (13.7, 22.7)
**Smoking status**									
Current smoker	1998-2009	5.5 (4.7, 6.3)	2009-2012	12.6 (8.5, 16.9)	2012-2018	-6.8 (-7.6, -6.1)	–	–	2.6 (1.9, 3.3)
Ex-smoker	1998-2009	13.8 (13.5, 14.1)	2009-2013	-9.7 (-10.3, -9.2)	2013-2018	1.8 (1.4, 2.2)	–	–	5.7 (5.5, 5.9)
Non-smoker	1998-2008	-13.4 (-13.4, -13.4)	2008-2011	-11.9 (-12.2, -11.6)	2011-2015	3.8 (3.6, 4.0)	2015-2018	-19.8 (-20.1, -19.4)	-11.0 (-11.1, -10.9)
Colorectal									
**Age, years**									
55	1998-2006	10.1 (9.0, 11.3)	2006-2009	3.7 (0.8, 6.7)	2009-2012	-15.0 (-17.9, -11.9)	2012-2018	3.8 (2.9, 4.7)	3.1 (2.4, 3.9)
65	1998-2007	3.7 (3.6, 3.9)	2007-2010	-0.7 (-1.4, 0.1)	2010-2015	-8.3 (-8.6, -8.1)	2015-2018	14.2 (13.5, 14.9)	1.4 (1.2, 1.5)
75	1998-2006	3.5 (3.2, 3.8)	2006-2009	0.9 (-0.3, 2.1)	2009-2013	-8.7 (-9.4, -8.1)	2013-2018	-0.7 (-1.1, -0.2)	-0.5 (-0.7, -0.2)
85	1998-2008	5.4 (5.2, 5.5)	2008-2012	-0.6 (-0.9, -0.2)	2012-2015	2.5 (1.8, 3.1)	2015-2018	-7.4 (-7.8, -6.9)	1.7 (1.6, 1.9)
**Gender**									
Men	1998-2008	0.4 (0.3, 0.4)	2008-2011	-1.7 (-2.0, -1.4)	2011-2015	-9.2 (-9.3, -9.0)	2015-2018	1.0 (0.7, 1.3)	-1.8 (-1.9, -1.8)
Women	1998-2006	9.4 (8.0, 10.8)	2006-2009	4.6 (0.7, 8.6)	2009-2014	-12.4 (-13.9, -11.0)	2014-2018	23.6 (21.5, 25.7)	5.3 (4.4, 6.2)
**Ethnicity**									
White									
Others	1998-2009	3.7 (3.6, 3.8)	2009-2012	-1.2 (-1.9, -0.5)	2012-2015	-11.6 (-12.5, -10.8)	2015-2018	14.2 (13.5, 14.8)	2.0 (1.8, 2.2)
**Deprivation**	1998-2010	-10.1 (-10.1, -10.1)	2010-2013	20.1 (19.8, 20.4)	2013-2016	-24.9 (-25.1, -24.7)	2016-2018	21.2 (20.8, 21.7)	-5.8 (-5.9, -5.8)
Least deprived									
Most deprived	1998-2008	-1.6 (-1.7, -1.5)	2008-2011	3.0 (2.4, 3.6)	2011-2015	-20.3 (-20.7, -19.9)	2015-2018	15.2 (14.4, 16.0)	-2.7 (-2.9, -2.6)
**BMI group, kg/m^2**	1998-2008	14.9 (14.3, 15.5)	2008-2011	6.5 (5.0, 8.0)	2011-2015	-18.2 (-19.1, -17.4)	2015-2018	51.3 (50.1, 52.6)	10.6 (10.2, 11.0)
18.5-24.9									
25.0-29.9	1998-2007	-9.6 (-10.0, -9.2)	2007-2015	6.7 (6.2, 7.3)	2015-2018	11.1 (9.6, 12.6)	–	–	-0.3 (-0.7, 0.0)
30.0-34.9	1998-2008	5.0 (4.9, 5.1)	2008-2011	-2.3 (-2.9, -1.8)	2011-2015	-15.9 (-16.3, -15.4)	2015-2018	12.1 (11.3, 12.9)	0.3 (0.2, 0.5)
≥35.0	1998-2009	0.8 (0.5, 1.0)	2009-2013	-8.0 (-9.1, -6.9)	2013-2018	-0.3 (-1.1, 0.5)	–	–	-1.3 (-1.6, -1.0)
**Smoking status**	1998-2012	1.7 (1.6, 1.8)	2012-2016	-7.0 (-7.7, -6.3)	2016-2018	46.6 (45.1, 48.2)	–	–	3.6 (3.4, 3.8)
Current smoker									
Ex-smoker	1998-2008	-1.4 (-1.5, -1.3)	2008-2012	8.1 (7.5, 8.6)	2012-2015	-17.4 (-18.3, -16.5)	2015-2018	30.2 (29.6, 30.9)	2.0 (1.8, 2.2)
Non-smoker	1998-2008	0.2 (0.0, 0.5)	2008-2015	-4.1 (-4.5, -3.8)	2015-2018	19.9 (18.7, 21.1)	–	–	1.4 (1.1, 1.6)

APC: Annual Percentage Change; **AAPC**: Average Annual Percentage Change. –: Not applicable. Different number of segments for each stratum (period 1 to 4) are identified by Joinpoint regressions**.**

ESM Table 8. Annual percentage change and average annual percentage change in cancer-specific mortality rates (four type 2 diabetes-related cancers)

	Period 1	APC, %	Period 2	APC, %	Period 3	APC, %	Period 4	APC, %	AAPC for whole period, %
Pancreas									
**Age, years**									
55	1998-2007	6.7 (6.4, 6.9)	2007-2010	0.1 (-0.9, 1.1)	2010-2014	-9.3 (-9.9, -8.7)	2014-2018	5.2 (4.5, 5.9)	2.0 (1.8, 2.2)
65	1998-2008	15.0 (14.1, 15.9)	2008-2012	-7.1 (-8.6, -5.6)	2012-2018	2.6 (1.9, 3.3)	–	–	6.5 (6.0, 7.0)
75	1998-2008	9.5 (9.2, 9.9)	2008-2014	-6.8 (-7.2, -6.5)	2014-2018	0.7 (-0.2, 1.7)	–	–	2.6 (2.4, 2.9)
85	1998-2008	12.3 (12.1, 12.6)	2008-2011	-1.6 (-2.4, -0.8)	2011-2015	4.3 (3.9, 4.7)	2015-2018	-3.0 (-3.4, -2.5)	6.1 (6.0, 6.3)
**Gender**									
Men	1998-2005	12.1 (11.5, 12.6)	2005-2008	7.9 (6.7, 9.1)	2008-2013	-9.7 (-10.0, -9.3)	2013-2018	-0.1 (-0.6, 0.4)	2.6 (2.3, 2.8)
Women	1998-2008	15.1 (14.8, 15.4)	2008-2012	-2.0 (-2.5, -1.5)	2012-2015	1.3 (0.3, 2.3)	2015-2018	-8.5 (-9.3, -7.8)	5.6 (5.4, 5.9)
**Ethnicity**									
White	1998-2008	21.5 (19.8, 23.3)	2008-2012	-8.2 (-10.3, -6.1)	2012-2018	1.3 (0.2, 2.3)	–	–	8.8 (7.9, 9.7)
Others	1998-2010	-3.2 (-3.3, -3.2)	2010-2014	-8.8 (-9.1, -8.5)	2014-2018	6.4 (6.1, 6.6)	–	–	-2.6 (-2.6, -2.5)
**Deprivation**									
Least deprived	1998-2006	27.2 (25.7, 28.7)	2006-2009	11.4 (9.5, 13.4)	2009-2014	-16.0 (-16.6, -15.4)	2014-2018	11.4 (10.1, 12.6)	9.5 (8.9, 10.0)
Most deprived	1998-2008	1.7 (1.5, 1.9)	2008-2012	8.0 (7.5, 8.6)	2012-2015	-7.2 (-8.1, -6.3)	2015-2018	17.2 (16.6, 17.9)	3.7 (3.5, 3.9)
**BMI group, kg/m^2**									
18.5-24.9	1998-2006	0.4 (0.1, 0.6)	2006-2010	18.4 (17.6, 19.2)	2010-2014	-15.0 (-15.6, -14.4)	2014-2018	-5.7 (-6.6, -4.8)	-0.9 (-1.1, -0.6)
25.0-29.9	1998-2008	23.5 (22.4, 24.5)	2008-2013	-10.5 (-11.2, -9.7)	2013-2018	3.2 (2.3, 4.2)	–	–	9.0 (8.4, 9.5)
30.0-34.9	1998-2009	6.7 (6.1, 7.4)	2009-2012	-11.2 (-14.5, -7.9)	2012-2018	6.3 (5.4, 7.1)	–	–	3.7 (3.1, 4.3)
≥35.0	1998-2010	0.8 (0.3, 1.3)	2010-2013	31.0 (27.0, 35.1)	2013-2018	-15.9 (-16.6, -15.1)	–	–	0.2 (-0.3, 0.8)
**Smoking status**									
Current smoker	1998-2008	36.1 (33.5, 38.8)	2008-2014	-8.9 (-9.6, -8.2)	2014-2018	2.9 (1.2, 4.7)	–	–	14.1 (13.0, 15.2)
Ex-smoker	1998-2008	6.5 (6.2, 6.7)	2008-2012	-7.6 (-8.2, -7.0)	2012-2015	10.0 (8.7, 11.3)	2015-2018	-9.6 (-10.4, -8.8)	1.5 (1.2, 1.7)
Non-smoker	1998-2008	11.6 (11.3, 11.9)	2008-2012	-10.3 (-10.9, -9.6)	2012-2015	7.7 (5.9, 9.4)	2015-2018	-2.9 (-3.9, -1.9)	4.1 (3.7, 4.4)
Liver									
**Age, years old**									
55	1998-2009	6.9 (6.6, 7.2)	2009-2013	17.5 (16.9, 18.1)	2013-2016	-12.5 (-13.3, -11.8)	2016-2018	-2.2 (-3.8, -0.6)	4.8 (4.5, 5.0)
65	1998-2015	5.8 (5.5, 6.1)	2015-2018	-18.6 (-21.8, -15.2)	–	–	–	–	1.7 (1.1, 2.4)
75	1998-2009	12.3 (11.4, 13.2)	2009-2013	-15.1 (-16.9, -13.2)	2013-2016	24.7 (18.7, 30.9)	2016-2018	-20.6 (-26.1, -14.7)	4.2 (3.1, 5.3)
85	1998-2009	5.8 (5.5, 6.1)	2009-2014	-1.5 (-2.0, -0.9)	2014-2018	5.6 (5.1, 6.2)	–	–	3.9 (3.7, 4.1)
**Gender**									
Men	1998-2009	4.4 (4.2, 4.7)	2009-2013	-15.2 (-16.1, -14.3)	2013-2016	17.3 (14.6, 20.1)	2016-2018	-14.7 (-17.3, -11.9)	-0.1 (-0.6, 0.4)
Women	1998-2010	32.8 (31.9, 33.8)	2010-2013	-19.1 (-20.1, -18.1)	2013-2016	34.9 (32.8, 37.0)	2016-2018	-34.5 (-36.6, -32.4)	15.2 (14.5, 15.8)
**Ethnicity**									
White	1998-2010	9.0 (8.6, 9.4)	2010-2013	-12.4 (-14.3, -10.4)	2013-2016	20.5 (17.8, 23.2)	2016-2018	-29.7 (-33.1, -26.1)	2.5 (1.8, 3.2)
Others	1998-2010	24.1 (23.1, 25.0)	2010-2013	-55.2 (-60.4, -49.3)	2013-2016	121.3 (-12.2, 457.3)	2016-2018	-41.8 (-57.3, -20.7)	7.7 (-5.1, 22.1)
**Deprivation**									
Least deprived	1998-2010	11.5 (11.4, 11.5)	2010-2013	4.3 (4.1, 4.5)	2013-2016	27.7 (27.6, 27.9)	2016-2018	-50.5 (-50.9, -50.1)	3.9 (3.8, 3.9)
Most deprived	1998-2007	29.9 (29.1, 30.7)	2007-2010	14.4 (13.7, 15.1)	2010-2014	-32.0 (-32.5, -31.4)	2014-2018	24.2 (23.3, 25.0)	11.0 (10.6, 11.3)
**BMI group, kg/m^2**									
18.5-24.9	1998-2015	18.9 (14.6, 23.3)	2015-2018	-37.8 (-62.1, 2.0)	–	–	–	–	7.9 (0.1, 16.2)
25.0-29.9	1998-2009	6.5 (6.3, 6.7)	2009-2012	-17.9 (-19.0, -16.8)	2012-2015	23.9 (21.7, 26.2)	2015-2018	-22.9 (-24.2, -21.5)	-0.2 (-0.6, 0.2)
30.0-34.9	1998-2018	5.5 (2.6, 8.4)	–	–	–	–	–	–	5.5 (2.6, 8.4)
≥35.0	1998-2008	12.3 (10.9, 13.6)	2008-2011	7.1 (4.1, 10.2)	2011-2014	-18.3 (-21.2, -15.3)	2014-2018	-3.5 (-5.7, -1.3)	3.1 (2.2, 4.1)
**Smoking status**									
Current smoker	1998-2009	-4.3 (-4.7, -3.9)	2009-2013	15.4 (13.4, 17.5)	2013-2016	-15.5 (-18.6, -12.4)	2016-2018	50.8 (46.6, 55.1)	2.0 (1.4, 2.7)
Ex-smoker	1998-2012	-5.2 (-5.3, -5.0)	2012-2016	12.3 (11.2, 13.5)	2016-2018	-31.1 (-34.2, -27.9)	–	–	-5.0 (-5.4, -4.5)
Non-smoker	1998-2010	21.3 (19.9, 22.6)	2010-2013	-27.2 (-30.2, -24.1)	2013-2016	28.7 (21.9, 35.9)	2016-2018	-42.3 (-51.7, -31.1)	5.2 (3.3, 7.2)
Gallbladder									

	Period 1	APC, %	Period 2	APC, %	Period 3	APC, %	Period 4	APC, %	AAPC for whole period, %
**Age, years old**									
65	1998-2013	-14.6 (-14.7, -14.6)	2013-2016	113.1 (112.1, 114.1)	2016-2018	-84.4 (-85.2, -83.6)	–	–	-17.4 (-17.8, -17.0)
75	1998-2010	-24.8 (-25.5, -24.0)	2010-2014	40.1 (15.4, 70.2)	2014-2018	-1.5 (-8.7, 6.2)	–	–	-10.1 (-13.5, -6.6)
85	1998-2012	-0.9 (-1.1, -0.8)	2012-2015	40.3 (38.6, 42.1)	2015-2018	-27.7 (-37.4, -16.4)	–	–	-0.4 (-2.4, 1.6)
Endometrium									
**Age, years old**									
55	1998-2009	-5.7 (-6.2, -5.1)	2009-2013	21.1 (17.9, 24.4)	2013-2018	-23.9 (-26.0, -21.7)	–	–	-6.0 (-6.8, -5.2)
65	1998-2007	14.1 (13.6, 14.6)	2007-2010	8.7 (7.6, 9.8)	2010-2014	-3.5 (-3.9, -3.0)	2014-2018	5.8 (5.5, 6.2)	7.9 (7.7, 8.2)
75	1998-2009	9.8 (9.2, 10.4)	2009-2013	-24.8 (-26.8, -22.8)	2013-2018	22.4 (20.6, 24.1)	–	–	4.6 (3.9, 5.3)
85	1998-2009	23.4 (22.9, 24.0)	2009-2013	1.0 (0.5, 1.4)	2013-2016	22.7 (22.1, 23.4)	2016-2018	-11.6 (-12.2, -11.0)	14.6 (14.3, 14.9)

Due to small numbers of events, stratified analyses were not possible for gallbladder and endometrial cancer mortality while rate for gallbladder cancer mortality in 55-year-old subjects was not estimable.

APC: Annual Percentage Change; **AAPC**: Average Annual Percentage Change. –: Not applicable. Different number of segments for each stratum (period 1 to 4) are identified by Joinpoint regressions**.**

ESM Figure 1. Study participants selection flowchart

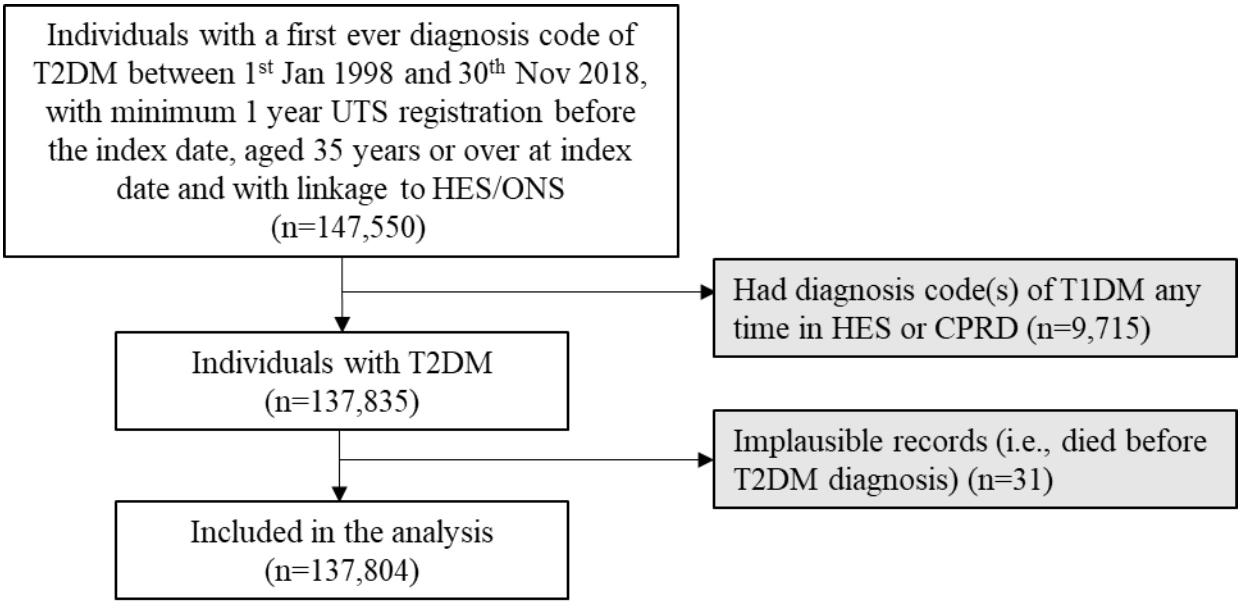

T2DM: type 2 diabetes; T1DM: type 1 diabetes; UTS: up-to-standard date; HES: Hospital Episodes Statistics; ONS: Office for National Statistics.

The index date was the first diagnosis date of type 2 diabetes.

RECORD checklist

	Item No.	STROBE items	Location in manuscript where items are reported	RECORD items	Location in manuscript where items are reported
**Title and abstract**					
	1	(a) Indicate the study's design with a commonly used term in the title or the abstract (b) Provide in the abstract an informative and balanced summary of what was done and what was found		RECORD 1.1: The type of data used should be specified in the title or abstract. When possible, the name of the databases used should be included.<br>RECORD 1.2: If applicable, the geographic region and timeframe within which the study took place should be reported in the title or abstract.<br>RECORD 1.3: If linkage between databases was conducted for the study, this should be clearly stated in the title or abstract.	1, 3
**Introduction**					
Background rationale	2	Explain the scientific background and rationale for the investigation being reported			5
Objectives	3	State specific objectives, including any prespecified hypotheses			5
**Methods**					
Study Design	4	Present key elements of study design early in the paper			6, 7
Setting	5	Describe the setting, locations, and relevant dates, including periods of recruitment, exposure, follow-up, and data collection			6, 7
Participants	6	*(a) Cohort study* - Give the eligibility criteria, and the sources and methods of selection of participants. Describe methods of follow-up<br>*(b) Cohort study* - For matched studies, give matching criteria and number of exposed and unexposed		RECORD 6.1: The methods of study population selection (such as codes or algorithms used to identify subjects) should be listed in detail. If this is not possible, an explanation should be provided.<br>RECORD 6.2: Any validation studies of the codes or algorithms used to select the population should be referenced. If validation was conducted for this study and not published elsewhere, detailed methods and results should be provided.<br>RECORD 6.3: If the study involved linkage of databases, consider use of a flow diagram or other graphical display to demonstrate the data linkage process, including the number of individuals with linked data at each stage.	6,7, ESM fig. 1
Variables	7	Clearly define all outcomes, exposures, predictors, potential confounders, and effect modifiers. Give diagnostic criteria, if applicable.		RECORD 7.1: A complete list of codes and algorithms used to classify exposures, outcomes, confounders, and effect modifiers should be provided. If these cannot be reported, an explanation should be provided.	6,7, Github ID: supingling
Data sources/ measurement	8	For each variable of interest, give sources of data and details of methods of assessment (measurement).<br>Describe comparability of assessment methods if there is more than one group			6, 7
Bias	9	Describe any efforts to address potential sources of bias			NA
Study size	10	Explain how the study size was arrived at			7, ESM fig. 1

Quantitative variables	11	Explain how quantitative variables were handled in the analyses. If applicable, describe which groupings were chosen, and why			6
Statistical methods	12	(a) Describe all statistical methods, including those used to control for confounding<br>(b) Describe any methods used to examine subgroups and interactions<br>(c) Explain how missing data were addressed<br>(d) *Cohort study* - If applicable, explain how loss to follow-up was addressed<br>(e) Describe any sensitivity analyses			7
Data access and cleaning methods		..		RECORD 12.1: Authors should describe the extent to which the investigators had access to the database population used to create the study population.<br>RECORD 12.2: Authors should provide information on the data cleaning methods used in the study.	6, 16
Linkage		..		RECORD 12.3: State whether the study included person-level, institutional-level, or other data linkage across two or more databases. The methods of linkage and methods of linkage quality evaluation should be provided.	6, 7
**Results**					
Participants	13	(a) Report the numbers of individuals at each stage of the study (*e.g.*, numbers potentially eligible, examined for eligibility, confirmed eligible, included in the study, completing follow-up, and analysed)<br>(b) Give reasons for non-participation at each stage.<br>(c) Consider use of a flow diagram		RECORD 13.1: Describe in detail the selection of the persons included in the study (*i.e.,* study population selection) including filtering based on data quality, data availability and linkage. The selection of included persons can be described in the text and/or by means of the study flow diagram.	8, ESM fig. 1
Descriptive data	14	(a) Give characteristics of study participants (*e.g.*, demographic, clinical, social) and information on exposures and potential confounders<br>(b) Indicate the number of participants with missing data for each variable of interest<br>(c) *Cohort study* - summarise follow-up time (*e.g.*, average and total amount)			8, Table 1
Outcome data	15	*Cohort study* - Report numbers of outcome events or summary measures over time			8, Table 2
Main results	16	(a) Give unadjusted estimates and, if applicable, confounder-adjusted estimates and their precision (e.g., 95% confidence interval). Make clear which confounders were adjusted for and why they were included<br>(b) Report category boundaries when continuous variables were categorized			8-11, Table 3, Table 4, Figure 1-4, ESM Table 1-8

		(c) If relevant, consider translating estimates of relative risk into absolute risk for a meaningful time period			
Other analyses	17	Report other analyses done—e.g., analyses of subgroups and interactions, and sensitivity analyses			8-11, Table 3, Table 4, Figure 1-4, ESM Table 1-8
**Discussion**					
Key results	18	Summarise key results with reference to study objectives			12
Limitations	19	Discuss limitations of the study, taking into account sources of potential bias or imprecision. Discuss both direction and magnitude of any potential bias		RECORD 19.1: Discuss the implications of using data that were not created or collected to answer the specific research question(s). Include discussion of misclassification bias, unmeasured confounding, missing data, and changing eligibility over time, as they pertain to the study being reported.	12, 13
Interpretation	20	Give a cautious overall interpretation of results considering objectives, limitations, multiplicity of analyses, results from similar studies, and other relevant evidence			12-15
Generalisability	21	Discuss the generalisability (external validity) of the study results			12-15,
**Other Information**					
Funding	22	Give the source of funding and the role of the funders for the present study and, if applicable, for the original study on which the present article is based			16
Accessibility of protocol, raw data, and programming code		..		RECORD 22.1: Authors should provide information on how to access any supplemental information such as the study protocol, raw data, or programming code.	16

Benchimol EI, Smeeth L, Guttmann A, Harron K, Moher D, Petersen I, Sørensen HT, von Elm E, Langan SM, the RECORD Working Committee. The REporting of studies Conducted using Observational Routinely-collected health Data (RECORD) Statement. *PLoS Medicine* 2015.

Checklist is protected under Creative Commons Attribution (CC BY) license.

Page numbers refer to the original, word document submission.
